# RSPO1, a potent inducer of pancreatic β cell neogenesis

**DOI:** 10.1016/j.xcrm.2025.102126

**Published:** 2025-05-07

**Authors:** Serena Silvano, Tiziana Napolitano, Magali Plaisant, Anette Sousa-De-Veiga, Hugo Fofo, Chaïma Ayachi, Benoit Allegrini, Samah Rekima, Estelle Pichery, Jérôme Becam, Valentin Lepage, Caroline Treins, Laura Etasse, Loan Tran, Julien Thévenet, Gianni Pasquetti, Julie Kerr-Conte, François Pattou, Paolo Botti, Arduino Arduini, Jacques Mizrahi, Benjamin Charles, Patrick Collombat

**Affiliations:** 1 DiogenX, 180 Avenue du Prado, 13008 Marseille, France; 2 University Nice Cote D’Azur, Inserm, CNRS, iBV, 06100 Nice, France; 3 iBV, Institut de Biologie Valrose, University Nice Sophia Antipolis, Centre de Biochimie, Parc Valrose, 28, Avenue Valrose, 06108 Nice Cedex 2, France; 4 Université Côte d'Azur, Inserm, C3M, Nice, France; 5 Aix-Marseille Université, CNRS, Laboratoire de Chimie Bactérienne, UMR 7283, Institut de Microbiologie de la Méditerranée, 31 Chemin Joseph Aiguier, 13009 Marseille, France; 6 University Lille, Inserm, CHU Lille, U1190 Translational Research for Diabetes, European Genomic Institute for Diabetes, EGID, 59000 Lille, France

**Keywords:** Rspo1, Wnt/β-catenin signaling, β cell replication, endocrine pancreas, islets of Langerhans, diabetes

## Abstract

Inducing the neogenesis of pancreatic insulin-producing β cells holds great promise for diabetes research. However, non-toxic compounds with such activities remain to be discovered. Herein, we report the identification of RSPO1, a key agonist of the Wnt/β-catenin pathway, as an inducer of β cell replication. Specifically, we provide evidence that RSPO1 promotes a significant increase in β cell neogenesis *in vitro*, *ex vivo*, and *in vivo*. Importantly, RSPO1 administration is sufficient to activate Wnt/β-catenin signaling in β cells and counter chemically induced or autoimmune-mediated diabetes. Similarly, an optimized analog of RSPO1, allowing for weekly administration, also prevents diabetes *in vivo*. Lastly, the treatment of transplanted human islets with RSPO1 induces a significant 2.78-fold increase in human β cell numbers in only 60 days, these cells being functional. Such activities of RSPO1 to promote β cell neogenesis could therefore represent an unprecedented hope in the continued search for diabetes alternative therapies.

## Highlights

• RSPO1 stimulates murine β cell replication via activation of the Wnt/β-catenin pathway
• RSPO1 administration increases β cell mass in NOD mice, thereby preventing hyperglycemia
• RSPO1 can maintain normoglycemia in two different models of chemically induced diabetes
• RSPO1 induces functional β cell mass increase in human tissues *ex vivo*

## Graphical abstract

**Figure:**
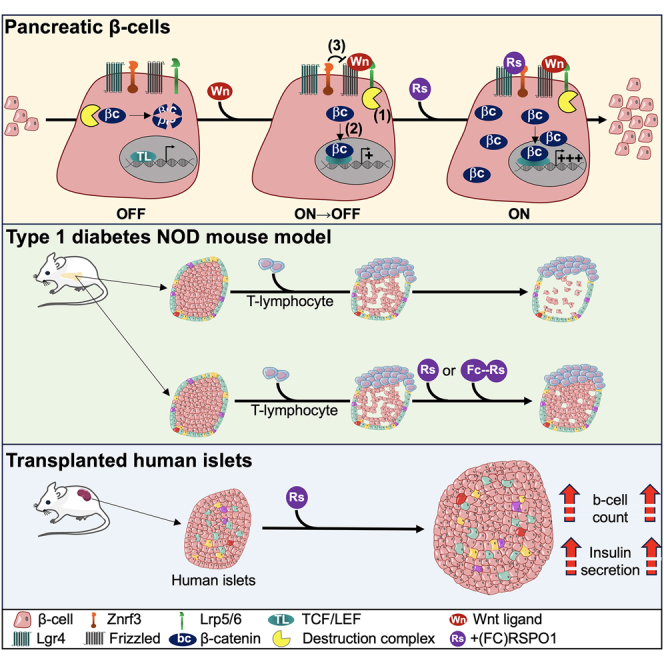

## Teaser

Silvano et al. show that RSPO1 stimulates pancreatic β cell proliferation via the activation of the Wnt/β-catenin signaling pathway. RSPO1 is shown to increase the β cell mass in NOD mice, thereby preventing hyperglycemia. Importantly, a similar RSPO1-induced functional β cell mass increase is observed in human tissues *ex vivo*.

## Introduction

The pancreas is an endoderm-derived digestive organ composed of an exocrine compartment and an endocrine portion. Within the exocrine compartment acinar cells produce, store, and secrete digestive enzymes. Scattered throughout the pancreas parenchyma, endocrine cells are organized in highly vascularized cell clusters termed islets of Langerhans. These contain five different hormone-producing cell subtypes classified as glucagon-producing α cells, insulin-producing β cell, somatostatin-producing δ cells, ghrelin-producing ε cells, and pancreatic polypeptide-producing (PP) cells.^1^^,^^2^ The main role of the endocrine pancreas is the regulation of glucose homeostasis. Accordingly, glucagon and insulin work coordinately by, respectively, increasing or decreasing blood glucose levels.^3^ This tightly controlled balance is severely impaired in diabetes mellitus (DM).

Type 1 diabetes mellitus is a multifactorial autoimmune disease characterized by the selective loss of pancreatic β cells, resulting in insufficient insulin production and chronic hyperglycemia. As of today, exogenous insulin supplementation remains the main approach allowing an acceptable control of blood glucose levels in type 1 diabetes (T1D) patients. However, although this approach saves lives, diabetic patients continue to suffer from long-term side effects, as exogenous insulin fails to reproduce the dynamic endogenous insulin production.^4^

The transplantation of islets of Langerhans represents a valid approach to restore endogenous insulin production. However, due to the shortage of donors and the associated life-long immunosuppression, this approach remains rarely used. Besides, while the differentiation of human embryonic or pluripotent stem cells into insulin-producing cells has recently made significant progresses,^5^^,^^6^ a certain degree of concern still surrounds stem cell-based therapies. Similarly, while insulin-producing cells could be also obtained by trans-differentiation of alternative pancreatic or non-pancreatic cell types,^7^^,^^8^^,^^9^^,^^10^^,^^11^^,^^12^^,^^13^^,^^14^ a possible generalized application remains distant. Thus, alternative therapies must be developed. Toward this goal, several studies demonstrated how different stimuli can strongly enhance either murine^15^^,^^16^^,^^17^ or human^18^ pancreatic β cell replication. In parallel, high-throughput screens and RNAi analyses have identified several compounds inducing human β cell replication.^18^^,^^19^ These agents include DIRK1A inhibitors, osteoprotegerin, denosumab, and SerpinB1, all molecules inducing β cell replication by acting on key pancreatic signaling pathways.^20^^,^^21^^,^^22^^,^^23^^,^^24^ However, while being promising, none of these compounds is currently used in a clinical context due to long-term toxicity, thus further emphasizing the need for alternative therapies.

Interestingly, the canonical Wnt signaling, also known as Wnt/β-catenin pathway, has been suggested for its putative involvement in β cell replication.^25^^,^^26^^,^^27^ This signaling cascade is highly conserved and mostly active during embryonic development to increase cell mass and organ size. Subsequently, the Wnt pathway is either down-regulated or switched off, except in restricted cell niches within highly regenerating tissues.^28^

Specifically, in the absence of Wnt ligands, or in presence of one or more Wnt antagonists, cytoplasmic β-catenin is continuously degraded by a group of proteins forming the so-called destruction complex, composed of different proteins (Axin, adenomatous polyposis coli [APC], and Disheveled) and kinases (CK1α and Gsk3). When the pathway is in such “OFF-state,” CK1α and Gsk3 selectively phosphorylate specific β-catenin residues, leading to its ubiquitination and proteasomal degradation. Conversely, upon interaction of Wnt ligands with their Frizzled receptor and co-receptors Lrp5 and 6, they induce their dimerization and a conformational change that triggers signal transduction.^29^ Subsequently, several components of the degradation complex are recruited at the plasma membrane, thus preventing β-catenin degradation. Consequently, the active non-phosphorylated form of β-catenin accumulates into the cytoplasm and eventually translocates into the nucleus to transactivate the transcription of the Wnt target genes.^28^ It is worth mentioning that the Wnt signaling is quickly down-regulated following the degradation of its main receptor Lrp5-6 by the E3 ubiquitin ligase zinc RING finger 3 (Znrf3). Importantly, R-spondin proteins have been showed to play a pivotal role in potentiating Wnt signaling upon interaction with both their specific receptors, leucine-rich repeat-containing G-protein coupled receptor (Lgr) 4, 5, or 6 and Znrf3, thereby preventing Znrf3-mediated Lrp receptor degradation.^30^

R-spondins belong to a family of highly evolutionary-conserved secreted peptides including four different members (Rspo1–4). Structurally, these are composed of an amino-terminal signal peptide sequence, two cysteine-rich furin-like (FU) domains, a thrombospondin type I repeat domain and a carboxy-terminal basic amino acid-rich domain.^31^ Importantly, the two cysteine-rich FU-like domains have been shown to play an essential role in the interaction with receptors and co-receptors.^32^^,^^33^ Interestingly, in a screen aiming at identifying new molecules able to trigger β cell replication (data not shown), we recently identified Rspo1. Herein, we report that treatment with human RSPO1 significantly increases β cell replication/number *in vitro*, *ex vivo*, and *in vivo*. Remarkably, long-term RSPO1 administration can prevent mice from developing hyperglycemia in different models of T1D. In this study, we provide evidence that the activation of the canonical Wnt signaling represents the main mechanism underlying the increased β cell replication. Importantly, we also demonstrate that treatment with RSPO1 increases the replication of adult human β cells, such results suggesting a possible previously unexplored application of RSPO1 or RSPO1-like compounds for the treatment of T1D patients.

## Results

### *Rspo1* expression in the pancreas is restricted to the acinar compartment

While a role of Rspo1 had been previously suggested in pancreas physiology,^34^^,^^35^^,^^36^ its expression and localization within this gland had been poorly investigated. To comprehensively address this question, and in the absence of commercially available working anti-Rspo1 antibodies, we resorted to quantitative reverse-transcription PCR (RT-qPCR) analyses. *Rspo1* transcripts were thus detected in the mouse pancreas as early as embryonic day (E)15.5 (Figure S1A). Interestingly, the *Rspo1* transcript content was found to increase after birth (post-natal day [P]0) with a peak at P6. Subsequently, *Rspo1* expression significantly decreased, returning to embryonic development levels. Thereafter, no fluctuation of *Rspo1* mRNA abundancy was detected at all adult ages analyzed.

**Figure 1:**
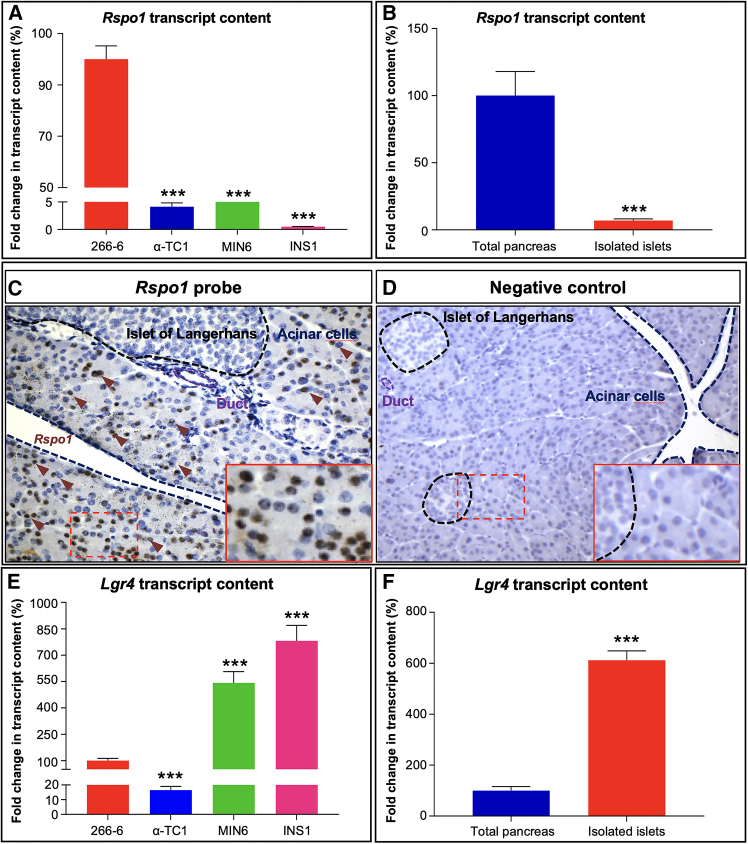
*Rspo1* and *Lgr4* expression and localization in the mouse pancreas (A) Determination of *Rspo1* transcript levels by RT-qPCR in pancreatic cell lines. (B) Assessment of *Rspo1* transcripts in whole pancreas versus isolated murine islet extracts. (C and D) *Rspo1* localization within the pancreas using RNAscope. 21-day-old WT pancreata labeled with a probe specifically recognizing the *Rspo1* transcript (C). Pancreatic section from the same animal labeled using a negative control probe (D). (E) RT-qPCR analyses of four different pancreatic cell lines to characterize *Lgr4* expression within the mouse pancreas. (F) Determination of *Lgr4* expression in whole pancreas and isolated mouse islets. All data shown represent mean ± SEM of *n* = 5. Results were considered significant if *p* < 0.0001 (∗∗∗∗), *p* < 0.001 (∗∗∗), *p* < 0.01 (∗∗), and *p* < 0.05 (∗) using a one-way ANOVA (A, E, and F) or a Student’s t test (B). See also Figure S1.

We subsequently sought to identify the cell type specifically expressing *Rspo1*. Toward this goal, we analyzed the mRNA levels of *Rspo1* in four different pancreatic cell lines: the 266-6 acinar cell line, the alpha-tumor cell line α-TC1, the mouse insulinoma β cell line MIN6, and the rat insulinoma β cell line INS-1 (Figure 1A). In addition, *Rspo1* expression was also evaluated in isolated islets of Langerhans and compared with whole pancreas extracts (Figure 1B). Interestingly, *Rspo1* was abundantly detected in the 266-6 acinar cell line and in whole pancreatic tissue, its expression being negligible in endocrine-derived cell lines and isolated islets of Langerhans (Figures 1A and 1B).

To further validate these results, and, again, due to the lack of commercial antibodies specifically recognizing Rspo1, we resorted to RNAscope to establish its localization within the pancreas. The analysis of tissue sections of 21-day-old wild-type (WT) pancreata revealed a strong signal in the acinar compartment, such expression lacking in endocrine and ductal cells (Figure 1C). To confirm the specificity of the signal observed, a probe targeting a gene not expressed in eukaryotic cells was used as negative control (Figure 1D). Taken together, our results indicate that *Rspo1* expression is restricted to the pancreatic acinar compartment and largely excluded from endocrine cells both *in vitro* and *in vivo*.

### *Lgr4* receptor transcripts are mostly detected in pancreatic β cells

Aiming to gain further insights into the putative role of Rspo1 within the pancreas, we assessed the expression of its three receptors, *Lgr4*, *5*, and *6*. Specifically, WT whole pancreata were analyzed by RT-qPCR at different embryonic and adult (st)ages. Interestingly, the results obtained revealed an expression of *Lgr4* in the developing pancreas as early as E15.5, its expression progressively increasing in a stepwise fashion from fetal development to adulthood. Conversely, *Lgr5* transcripts were solely detected until birth. Finally, *Lgr6* mRNAs were never detected in both the developing and adult organ, thus suggesting that this receptor is not required for Rspo1-mediated Wnt signaling in the mouse pancreas (Figure S1B).

Having established that Lgr4 is the sole Lgr receptor expressed in adult mice, we aimed to define its localization. Yet again, due to the lack of working commercial antibodies and RNAscope probes specifically recognizing Lgr4, we resorted to quantitative assays to evaluate its expression in four different cell lines and in isolated islets of Langerhans. *Lgr4* expression was thus abundantly detected in MIN6 and INS-1 β cell lines, its expression being lower or totally absent in the acinar 266-6 line and in α-TC1 cells (Figure 1E). Accordingly, *Lgr4* expression in WT isolated islet of Langerhans was found to be six times higher when compared to the whole organ (Figure 1F). Altogether, our results indicate that, throughout adulthood, *Lgr4* is more abundantly expressed in pancreatic β cells as compared to the exocrine compartment.

### RSPO1 treatment induces an increase of pancreatic β cell number *in vitro*, *ex vivo*, and *in vivo*

**Figure 2:**
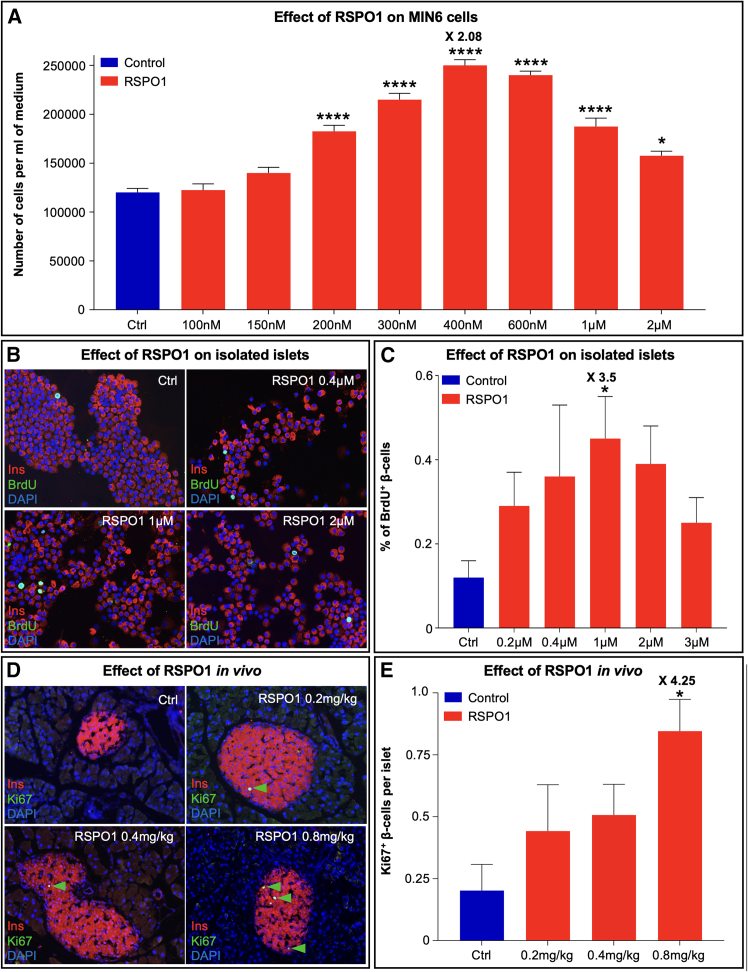
RSPO1 induces β cell replication in MIN6 cells, in isolated islets, and in adult WT mice (A) Assessment of MIN6 cell number upon 24-h incubation with increasing doses of native RSPO1 or saline. (B) Representative photographs of pancreatic islets isolated from WT adult mice incubated with either RSPO1 (at 0.4, 1, or 2 μM) or saline. Aiming to label replicating cells, isolated islets were co-incubated with BrdU during the last 24 h and then stained for insulin (red) and BrdU (green). (C) Quantification of the percentage of BrdU^+^ β cells in control islets and islets incubated for 72 h with increasing doses of RSPO1. (D) Pancreatic sections obtained from adult WT mice administered intraperitoneally for 5 consecutive days with different doses of RSPO1 and stained for insulin (red) and Ki67 (green). (E) Quantitative assessment of Ki67^+^ cells per islet after 5 consecutive RSPO1 administrations. All data shown represent mean ± SEM of *n* = 5. Results were considered significant if *p* < 0.0001 (∗∗∗∗), *p* < 0.001 (∗∗∗), *p* < 0.01 (∗∗), and *p* < 0.05 (∗) using one-way ANOVA. See also Figure S2.

Having established the expression of *Rspo1* and its main receptor *Lgr4* within the pancreas, we sought to determine the role of this secreted protein in pancreatic β cells, as previous reports were controversial.^34^^,^^35^^,^^36^ We therefore incubated MIN6 cells with increasing doses of the human RSPO1 molecule. Importantly, cell quantification revealed a potent and significant dose-dependent increase in β cell count, with a 2.08-fold peak increase (versus controls) at 400 nM in only 24 h (Figure 2A). To verify whether RSPO1 would also promote the replication of adult murine β cells, which are less prone to undergo replication as compared to immortalized β cell lines,^37^^,^^38^^,^^39^ we incubated WT mouse islets *ex vivo* with different concentrations of RSPO1 for 72 h. Interestingly, our observations revealed that RSPO1 triggered the replication of a significant number of β cells (Figure 2B). Quantitative analyses of BrdU^+^/Insulin^+^ cells confirmed this increase with a dose-dependent response and a peak at 1 μM, outlining a 3.5-fold increase when compared to controls (Figure 2C). To complete our analysis, we have performed a quantitative analysis of pancreatic α cells and the Ki67+ exocrine cells (normalized on the total tissue area) and thereby determined no significant difference when comparing treated versus non-treated conditions (Figures S2A and S2B). Of note, a non-significant 1.4-fold increase was noted in the number of proliferating exocrine cells in mice administered with 0.8 mg/kg of native RSPO1. One should consider that the number of proliferating exocrine cells is extremely low in normal conditions, such trend toward an augmented cell count being therefore negligible. Accordingly, the pancreas weight of these mice was found to be unchanged between experimental groups (Figure S2C).

To further corroborate the ability of RSPO1 to increase insulin-producing cell replication, a short-term *in vivo* assessment was employed. Specifically, 8-week-old WT mice were daily injected intraperitoneally over 5 consecutive days with 0.2, 0.4, or 0.8 mg/kg of RSPO1. Quantitative analyses of pancreatic sections from control and treated animals stained with anti-insulin and anti-Ki67 antibodies (Figure 2D) revealed that RSPO1 promoted β cell replication *in vivo*, as outlined by the significant 4.25-fold increase in Ki67+/Insulin+ cell number observed at the dose of 0.8 mg/kg (Figure 2E). Taken together, our results not only confirm that RSPO1 can robustly increase the number of β cells *in vitro* but also provide evidence that this secreted protein can significantly enhance the replication of adult murine β cells both *ex vivo* and *in vivo*.

### RSPO1 induces pancreatic β cell proliferation by activating the Wnt/β-catenin signaling pathway

**Figure 3:**
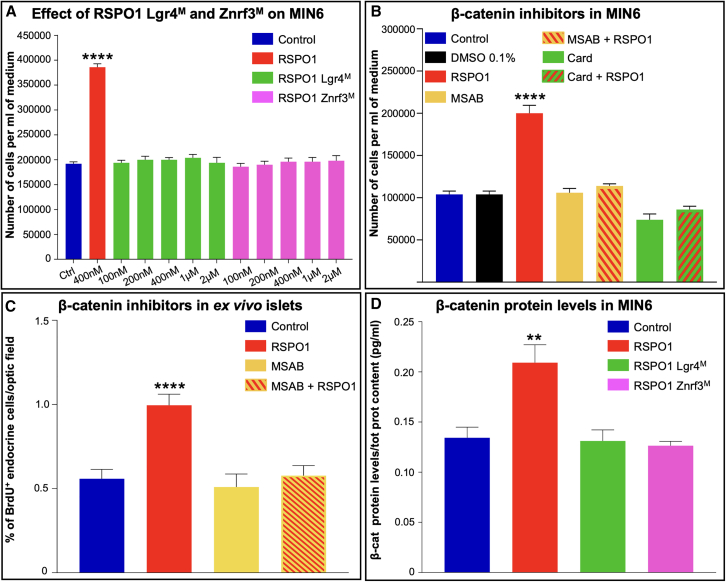
RSPO1 stimulates β cell replication via the activation of Wnt signaling The putative activation of the canonical Wnt signaling pathway upon RSPO1 incubation was assessed *in vitro*. (A) MIN6 cell number quantification upon incubation for 24 h with increasing doses of two RSPO1 mutants Lgr4^M^ and Znrf3^M^. (B) MIN6 cell number assessment upon a 24-h treatment with saline, 0.1% DMSO, 100 nM MSAB, or 500 nM cardamonin and co-incubation of MIN6 cells with either MSAB or cardamonin and native RSPO1 at 400 nM. (C) Quantification of BrdU^+^ cells in *ex vivo* murine islets incubated for 72 h with saline, 100 nM MSAB, 1 μM RSPO1, or a combination of the 2. (D) β-catenin protein levels upon RSPO1 treatment assessed by ELISA following a 3-h incubation with saline, native RSPO1, RSPO1 Lgr4^M^, or RSPO1 Znrf3^M^ at 400 nM. Data shown represent mean ± SEM of *n* = 5. Results were considered significant if *p* < 0.0001 (∗∗∗∗), *p* < 0.001 (∗∗∗), *p* < 0.01 (∗∗), and *p* < 0.05 (∗) using one-way ANOVA. See also Figure S3.

R-spondin proteins have been repeatedly shown to act as key potentiators of the Wnt/β-catenin signaling upon interacting with their specific receptors Lgr4, 5, or 6 and co-receptor Znrf3.^30^ Of note, the accurate reconstruction of the Lgr4/Znrf3-Rspo1 crystal structures allowed the identification of the specific Rspo1 amino acids involved in the interaction with its receptors.^32^^,^^33^^,^^40^ Aiming to ascertain whether the enhanced proliferation observed in MIN6 cells upon RSPO1 incubation was the consequence of the activation of the canonical Wnt signaling upon binding to Lgr4 and/or Znrf3, we combined different approaches. At first, we generated two RSPO1 analogs carrying aminoacidic mutations into the binding site with its receptors. More specifically, RSPO1 Lgr4^M^ carried a single substitution (F106A), preventing the binding of RSPO1 to Lgr4.^32^^,^^40^ Similarly, RSPO1 Znrf3^M^ carried the R66A and Q71A substitutions to abolish the interaction with Znrf3.^33^ To validate our mutants, and in absence of a working binding assay, we resorted to HEK293-STF (SuperTopFlash) cells, a luciferase reporter line that responds to Wnt signaling activation by expressing firefly luciferase. Interestingly, no luciferase expression was measured upon incubation of these cells with our RSPO1 mutants as compared to native RSPO1 (Figure S3). Having established that both mutants do not activate Wnt signaling, we successively incubated MIN6 cells with increasing concentrations of Lgr4^M^ or Znrf3^M^ analogs for 24 h and performed cell quantification. Interestingly, both molecules did not increase the β cell count at any of the concentrations tested when compared to native RSPO1 (Figure 3A) thus confirming that RSPO1 needs to interact with both receptors to induce β cell neogenesis.

To further establish a role of RSPO1 as inducer of β cell replication via Wnt signaling, we made use of two β-catenin inhibitors, MSAB (Methyl 3-{[(4-methylphenyl)sulfonyl]amino}benzoate) and cardamonin.^41^^,^^42^ These potent molecules selectively interact with cytoplasmic β-catenin and promote its degradation, thereby inhibiting the subsequent transactivation of target genes. To achieve our goal, we first incubated MIN6 cells with DMSO, MSAB, or cardamonin alone to rule out any toxicity of these compounds (Figure 3B). Subsequently, β cells were incubated with 400 nM RSPO1 (such dose allowing for the highest increase in β cell number; see Figure 2A) alone or in combination with either MSAB or cardamonin. Interestingly, the quantification of MIN6 cells confirmed the significant increase in cell number upon RSPO1 treatment and concomitantly showed no effect of this molecule when co-incubated with the two β-catenin inhibitors (Figure 3B). Furthermore, in an effort to demonstrate that such molecular mechanism would also underline the increased proliferation observed in mice, we incubated *ex vivo* WT mouse islets with RSPO1, MSAB, or a combination of them. Comparably, a remarkable and significant increase in BrdU^+^ endocrine cells was observed upon sole incubation with RSPO1, such effect being entirely abolished upon co-treatment with MSAB (Figure 3C).

In the light of these results, demonstrating the requirement of β-catenin for RSPO1-mediated β cell neogenesis, we performed an ELISA to quantify β-catenin protein levels in MIN6 cells incubated (or not) with RSPO1, Lgr4^M^, or Znrf3^M^ analogs. According to our previous observations, β-catenin was found to be significantly increased upon RSPO1 incubation. Conversely, RSPO1 Lgr4^M^ and Znrf3^M^ did not elicit such β-catenin accumulation (Figure 3D). Collectively, these findings provide evidence of the involvement of the canonical Wnt signaling pathway in RSPO1-mediated β cell neogenesis.

### Long-term RSPO1 administration protects mice from developing hyperglycemia in different *in vivo* models of T1D

As previously mentioned, T1D is a chronic metabolic disease characterized by the selective immune-mediated loss of pancreatic insulin-producing cells. However, such β cell loss is a slow-evolving process,^43^ and patients with long-standing disease still retain a percentage of functional β cells.^44^^,^^45^ We therefore wondered whether RSPO1 could enhance endogenous β cell replication in T1D context using different approaches.

**Figure 4:**
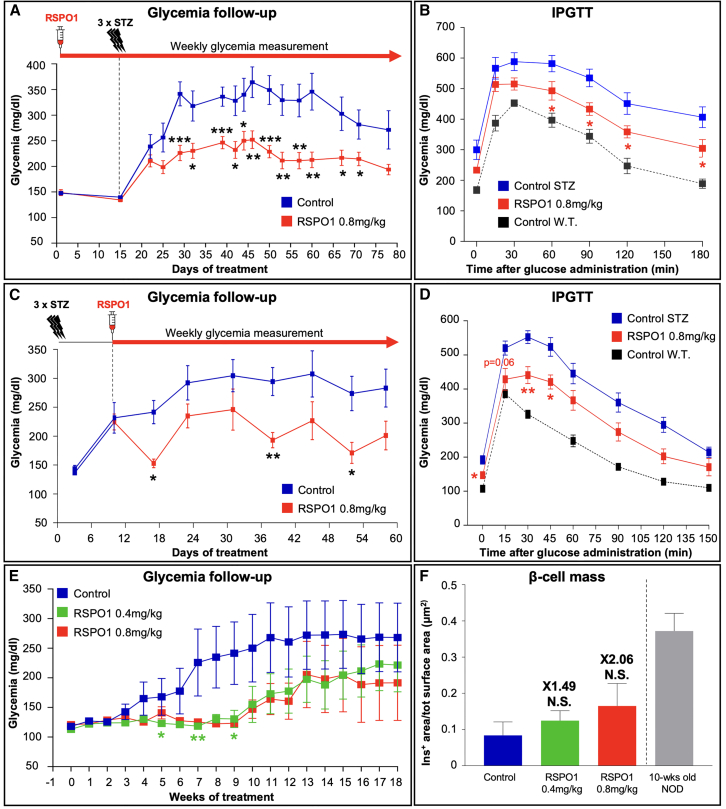
RSPO1 administration protects mice from developing hyperglycemia in different T1D models (A) Glycemia follow-up of two-month-old WT mice administered intraperitoneally for 80 consecutive days with either saline or 0.8 mg/kg of RSPO1 and co-treated with 50 mg/kg of streptozotocin (STZ) from day 16 to day 18 (*n* = 10). (B) IPGTT performed following 74 days of RSPO1 (or saline) treatment (*n* = 10). (C) Glycemia follow-up of six-week-old WT mice first administered for 3 consecutive days (1–3) with 50 mg/kg of STZ and subsequently injected daily with either saline or RSPO1 at 0.8 mg/kg once their glycemia reached 250 mg/dL (approximately at day 7) (*n* = 10). (D) IPGTT performed 63 days after first STZ administration (*n* = 10). (E) Weekly monitoring of random glycemia in 10-week-old NOD females injected intraperitoneally daily for 18 weeks with either saline or 0.4 or 0.8 mg/kg of RSPO1 (*n* = 15 in control group, *n* = 10 to 13 in treated groups). (F) Quantification of the whole β cell mass in NOD mice administered daily with either saline or 0.4 or 0.8 mg/kg of native RSPO1. The β cell mass of 10-week-old NOD females was used to evaluate the insulin^+^ area at the beginning of the study (*n* = 15 in control group, *n* = 10 to 13 in treated groups). All data shown represent mean ± SEM. Results were considered significant if *p* < 0.0001 (∗∗∗∗), *p* < 0.001 (∗∗∗), *p* < 0.01 (∗∗), and *p* < 0.05 (∗) following a two-way ANOVA (A–D); an unpaired Student’s t test or Mann-Whitney test (B), a mixed-effect model (restricted maximum likelihood, REML) with a Dunnett’s comparison test (C); and a one-way ANOVA, a Mann-Whitney test, or a Kruskal-Wallis test (E). See also Figures S4 and S5.

In the first study, WT mice were pre-treated daily for 15 days with 0.8 mg/kg of RSPO1 and subsequently administered for 3 consecutive days (16–18) with 50 mg/kg of streptozotocin (STZ), daily RSPO1 administration being maintained until sacrifice (Figure 4A). The main goal of this approach was to evaluate whether RSPO1 treatment could protect mice from developing STZ-induced β cell loss. Expectedly, STZ administration induced a severe hyperglycemia already 10 days after STZ injection in saline-treated animals. Importantly, RSPO1-treated mice remained within a relatively normoglycemic range throughout the entire treatment, displaying significantly lower glycemia when compared to age-/sex-matched controls (Figure 4A). Of note, the monitoring of the body weight outlined a slight increase, albeit not significant, in treated animals (Figure S4A). Additionally, an intraperitoneal glucose tolerance test (IPGTT) was performed on day 74 to determine whether RSPO1 treatment could also lead to an ameliorated glucose handling. Accordingly, the analysis of the glycemic curves showed that animals injected daily with RSPO1 displayed a significantly lower glycemic peak when compared to controls (Figure 4B), as confirmed by the assessment of the area under the curve (AUC) of the two groups (Figure S4B).

In the second study, we aimed at assessing whether daily RSPO1 administration could also revert STZ-induced hyperglycemia once the animals were readily diabetic. Toward this goal, WT mice were first injected for 3 consecutive days with 50 mg/kg of STZ. Subsequently, RSPO1 intraperitoneal daily treatment was started on day 7, when all animals had reached a glycemia of at least 250 mg/dL. Importantly, weekly measurement of random-fed blood glucose demonstrated that, while mice exclusively treated with saline became overtly hyperglycemic, animals injected daily with RSPO1 not only did not develop severe hyperglycemia but displayed significantly lower blood glucose levels when compared to control animals (Figure 4C). Interestingly, at different time points, their glycemia was found to be lower than on day 7 (before initiating RSPO1 treatment), suggesting that RSPO1 can revert/mitigate the STZ-induced increase in blood glucose. Of note, the body weight monitoring showed a slight increase, albeit not significant, in treated animals (Figure S4C). Additionally, an IPGTT performed on day 63 revealed that animals which received a daily dose of RSPO1 exhibited an improved glucose tolerance as compared to controls (and as confirmed by the analysis of their AUC; Figure S4D), with a significantly reduced glycemic peak and a faster restoration to fasting glycemia levels (Figure 4D).

To further define the potential of RSPO1 in an autoimmune T1D-like context, we selected non-obese diabetic (NOD) mice, which spontaneously develop diabetes as result of leukocytic infiltration within the pancreatic islets. In this study, only females were used, as they classically display a higher diabetes incidence when compared to males. Specifically, 10-week-old female were daily administered for 18 weeks with either saline or 2 different doses (0.4 or 0.8 mg/kg) of RSPO1. Interestingly, the weekly monitoring of random-fed blood glucose revealed that, while saline-treated animals became overtly hyperglycemic already by week 7, mice injected daily with RSPO1 displayed a significantly lower glycemia and remained within a normoglycemic range for more than 18 weeks (Figure 4E). Accordingly, the diabetes incidence (the percentage of mice displaying a glycemia >250 mg/dL in each experimental group) was found to be considerably decreased in treated mice (Figure S4E). It is worth noting that, even following such an 18-week-long daily treatment with RSPO1, the animals appeared healthy and indistinguishable from their saline-administered counterparts, despite a slight decrease in the body weight of treated animals as compared to controls (Figure S4F). Accordingly, the measurement of the pancreas weight (Figure S4G), as well as the gross examination of their extra-pancreatic organs at sacrifice, did not reveal any abnormality. Interestingly, a macroscopical evaluation performed on pancreatic sections revealed that mice treated daily with RSPO1 (Figures S5B and S5C) presented a greater β cell mass compared to the saline-injected controls (Figure S5A). Such increase seemed to be dose related as mice injected with 0.4 mg/kg of RSPO1 (Figure S5B) displayed a lower number of pancreatic islets when compared with NOD mice administered daily with 0.8 mg/kg of this Wnt agonist (Figure S5C). Accordingly, the quantification of the total insulin area confirmed that mice injected with either 0.4 or 0.8 mg/kg of RSPO1, respectively, exhibited a 1.49- and 2.06-fold increase compared to control animals (Figure 4F). Importantly, similar results were obtained by normalizing the total insulin area on the pancreas weight (Figure S4H). In essence, our data provide evidence that daily RSPO1 treatment can maintain glycemia in a physiological range in two different models of STZ-induced diabetes. Additionally, daily administration of this molecule protects NOD mice from developing hyperglycemia by increasing their β cell mass.

### Weekly administration of an FC-coupled RSPO1 analog protects NOD mice from developing hyperglycemia and increases their β cell mass

In an effort to optimize RSPO1 activities, we generated an engineered version of RSPO1 by coupling it with an immunoglobulin G (IgG) fragment crystallizable (FC) domain (FC-RSPO1). This approach is nowadays widely used in drug development, as it allows to greatly extend protein half-lives, thus enhancing their therapeutic potency without altering their main structure and functions.^46^

**Figure 5:**
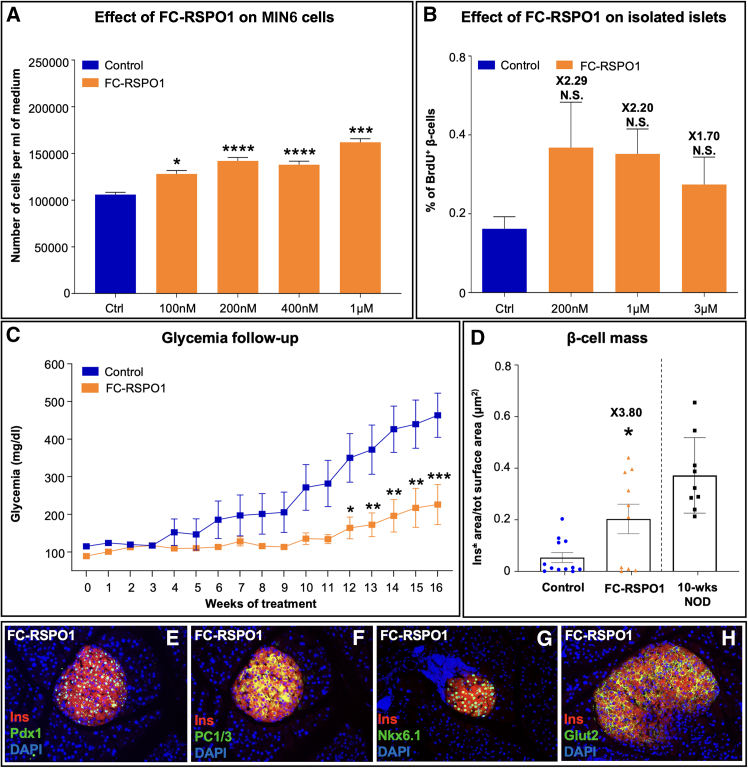
An FC-coupled RSPO1 induces pancreatic β cell neogenesis *in vitro*, *ex vivo*, and *in vivo* (A) Assessment of MIN6 cell number upon 24-h incubation with saline or increasing doses of FC-coupled RSPO1 protein (*n* = 5). (B) Quantification of the percentage of BrdU+ cells in saline-treated islets and islets incubated for 72 h with 200 nM or 1 or 3 μM FC-RSPO1 (*n* = 5). (C) Weekly monitoring of random glycemia in 10-week-old NOD females injected intraperitoneally weekly for 18 weeks with either saline or 2.4 mg/kg of FC-RSPO1 (*n* = 12 in control group, *n* = 10 in treated group). (D) Quantification of the whole β cell mass in NOD mice weekly administered with either saline or 2.4 mg/kg of FC-coupled RSPO1. The β cell mass of 10-week-old NOD females was used to evaluate the insulin^+^ area at the beginning of the study (*n* = 12 in control group, *n* = 10 in treated group). (E–H) Pancreatic sections from FC-RSPO1-treated NOD mice stained for the β cell markers Pdx1 (E), PC1/3 (F), Nkx6.1 (G), and Glut2 (H). All data shown represent mean ± SEM. Results were considered significant if *p* < 0.0001 (∗∗∗∗), *p* < 0.001 (∗∗∗), *p* < 0.01 (∗∗), and *p* < 0.05 (∗) following a one-way ANOVA (A and B); a one-way ANOVA, a Mann-Whitney test, or a Kruskal-Wallis test (C); or an unpaired Student’s t test (D).

To evaluate the ability of FC-RSPO1 to enhance pancreatic β cell proliferation, we first incubated MIN6 cells with increasing concentrations of FC-RSPO1 and performed manual counting. Interestingly, cell quantification assessed after 24 h of incubation revealed a significant increase in MIN6 cell number (Figure 5A).

We pursued our analyses by evaluating FC-RSPO1 proliferative capacities in *ex vivo* islets from WT mice. As formerly described, isolated islets were incubated over 72 h with 3 different doses of FC-RSPO1 and co-incubated for the last 24 h with bromodeoxyuridine (BrdU). Quantification analyses of double Ins+/BrdU+ cells, although not showing statistical significance, partially support the proliferative capacities of this FC-coupled RSPO1, which was found to increase adult β cell replication 2.29, 2.20, and 1.70 times when incubated at 0.2, 1.0, and 3.0 μM, respectively (Figure 5B). Interestingly, these data clearly show that coupling our native protein with an FC domain does not alter its function *in vitro* and *ex vivo*.

We then evaluated whether the administration of this FC-linked analog could be beneficial in a diabetic context. To address such question, NOD females were treated with FC-RSPO1 over 16 weeks. Importantly, considering that the FC domain significantly increases protein stability, FC-RSPO1 was only administered once a week instead of daily. For comparative “mole to mole” purpose, we used a 3-fold increased dose (2.4 mg/kg), when compared to RSPO1 (0.8 mg/kg), as the addition of an FC domain to RSPO1 results in a 3-fold increase in molecular weight. Strikingly, weekly measurement of random-fed glycemia revealed that, while control NOD mice gradually developed hyperglycemia, mice administered weekly with FC-RSPO1 remained within a normoglycemic range throughout the entire treatment (Figure 5C). Accordingly, quantitative assessment of the insulin^+^ area showed a significant 3.8-fold increase in the β cell mass of FC-RSPO1-treated NOD mice when compared to saline-injected controls (Figure 5D). To complete our characterization, we performed immunohistochemical analyses to assay the expression of *bona fide* β cell markers in FC-RSPO1-treated NOD mice (Figures 5E–5H, controls were not included due to the loss of β cells and the altered phenotype of the remaining ones). Interestingly, the majority of insulin-producing cells were found to express Pdx1 (involved in insulin secretion; Figure 5E), the prohormone convertase 1/3 (required to mature insulin; Figure 5F), the homeobox protein Nkx6.1 (involved in insulin secretion; Figure 5G), and the glucose transporter Glut2 (acting as a glucose sensor; Figure 5H), thereby further suggesting that neo-generated β cells displayed a β-like cell phenotype. Expectedly, a small percentage of insulin-positive cells were found to be negative for the aforementioned markers, most likely due to ongoing β cell death. Collectively, these data show that coupling RSPO1 with an IgG FC domain does not alter its activities *in vitro* and *ex vivo*. Furthermore, by increasing its half-life, we demonstrate a considerable amelioration of its therapeutic efficacy, as weekly administration appears to be remarkably efficacious in preventing NOD mice from spontaneously developing hyperglycemia.

### Long-term RSPO1 administration enhances adult human β cell proliferation and function

Aiming to determine whether RSPO1 would also efficiently stimulate the proliferation of human β cells, we transplanted human islets under the kidney capsule of immunodeficient RAG12N2 mice. This approach is nowadays widely used to monitor human islets over a prolonged period of time, as the islets are well integrated within the tissue, are highly vascularized, and secrete insulin in a glucose-stimulated fashion. Following transplantation, immunocompromised mice were administered daily with saline or RSPO1 (0.4 or 0.8 mg/kg) for 60 days. Weekly measurement of body weight did not show any significant difference between controls and treated mice (Figure S6A). Conversely, regular monitoring of random glycemia outlined significantly lower values in treated mice when compared to their control counterparts (Figure S6B).

**Figure 6:**
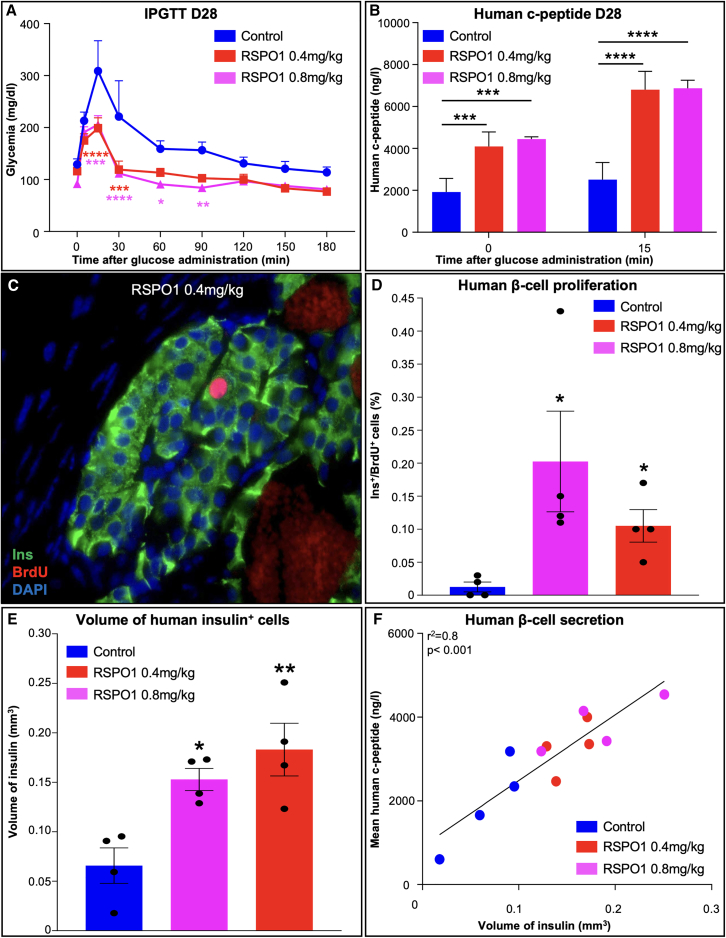
The administration of RSPO1 to immunodeficient mice transplanted with human islets induces functional human β cell neogenesis (A) IPGTT on transplanted RAG12N2 mice performed 28 days following daily saline or RSPO1 treatment initiation (0.4 and 0.8 mg/kg). (B) Assessment of human c-peptide circulating levels in control and RSPO1-treated mice at basal level and 15 min after glucose administration (*n* = 4). (C) Representative image of an immunohistochemical assay performed on grafted human islets following 60 days of RSPO1 administration (0.4 mg/kg) and labeling insulin in green and BrdU in red. (D) Quantitative analyses showing the percentage of human BrdU^+^/Ins^+^ double-positive cells in control grafts and in grafts obtained from mice treated with either 0.4 or 0.8 mg/kg of RSPO1. (E) Immunohistochemical quantification of the total insulin volume (mm^3^) within the transplants of control RAG12N2 mice and mice treated daily with either 0.4 or 0.8 mg/kg of RSPO1. (F) Correlation between the mean of the human c-peptide secretion (*y* axis) and the β cell mass (volume of insulin, *x* axis) calculated per animal. All data shown in (A), (B), (D), and (E) represent mean ± SEM of *n* = 4. Results were considered significant if *p* < 0.0001 (∗∗∗∗), *p* < 0.001 (∗∗∗), *p* < 0.01 (∗∗), and *p* < 0.05 (∗) following a two-way ANOVA (A and B) or a one-way ANOVA (D and E). See Figure S6.

To establish whether RSPO1 treatment would influence glucose metabolism, we performed an IPGTT 28 days post-treatment. Interestingly, RSPO1-treated mice displayed a significantly improved glucose tolerance, with a reduced glycemic peak and a return to normoglycemia only 30 min after the administration of a glucose bolus (Figure 6A). Accordingly, the concomitant assessment of human c-peptide serum levels revealed a significantly increased secretion of this metabolite upon RSPO1 administration, at both basal level and 15 min post-glucose stimulation (Figure 6B).

As previously demonstrated, RSPO1 administration strongly enhances the proliferation of murine pancreatic β cells. Aiming to determine whether this molecule could also induce the replication of adult human β cells, we performed phenotypical analyses and quantified endocrine cell proliferation within the kidney transplant 60 days post-treatment initiation. To evaluate cell division, we relied on BrdU incorporation analyses, following 7 days of BrdU supplementation prior to sacrifice. Interestingly, quantitative analyses of the BrdU^+^/Ins^+^ double-positive cells revealed a striking 5-to-10-fold increase in human β cell proliferation in RSPO1-treated mice (Figures 6C and 6D), suggesting an induction of β cell replication even after more than 50 days of daily treatment (BrdU labeling being performed only 7 days before sacrifice). Accordingly, the assessment of the total human insulin-producing cell volume showed a significant 2.32- and 2.78-fold increase in mice treated for 60 days with 0.4 and 0.8 mg/kg of RSPO1, respectively (Figure 6E).

To complete our investigations, we evaluated the direct relationship between the human β cell mass and its insulin secretion capabilities. Interestingly, correlation analyses demonstrated that control mice, displaying the lowest β cell mass, also secreted lower amounts of human c-peptide. Conversely, RSPO1-treated animals exhibited a significantly higher β cell mass and a remarkably enhanced secretion of human c-peptide (Figure 6F), hence providing evidence that the newly generated human β cells are metabolically functional. Altogether, our data clearly demonstrate a potent ability of RSPO1 in inducing human functional β cell neogenesis, thereby opening new avenues in the context of diabetes research.

## Discussion

Although accounting for only 2%–3% of the whole pancreatic mass, the endocrine compartment can be considered as the pulsating heart of glucose homeostasis. Hence, it represents the focal point of the numerous researches. In T1D context, pancreatic β cells represent the target of the immune system, which mistakenly and progressively destroys almost 90% of these, thereby inducing an insulin shortage and a consequent increase of blood glucose levels. Importantly, despite the life-saving role of exogenous insulin administration, T1D patients still display a shortened life expectancy and an altered quality of life, thereby highlighting the need for alternative therapeutic strategies to efficiently replenish/protect the β cell pool.

In the present work, we identified RSPO1 as a potent inducer of pancreatic β cell neogenesis.

Previous reports were unclear concerning the putative function of Rspo1 in β cells, some reports suggesting a negative role on β cell replication and other ones arguing the opposite.^34^^,^^35^ Thus, aiming to provide conclusive evidence, we tested RSPO1 capabilities in different contexts. We thereby demonstrate that RSPO1 promotes a dose-dependent and efficient increase in β cell number *in vitro*. Of note, a bell-shape-like curve was observed at higher concentrations: the exact reason of this decreased capability to induce β cell neogenesis beyond a certain threshold is unclear and would require further investigations. However, one should consider that MIN6 are immortalized cells, which are artificially proliferating. Therefore, despite remaining an extremely useful and reliable tool for preliminary investigations, they do not fully recapitulate the real β cell physiology. To address this limitation, we combined these *in vitro* studies with an *ex vivo* approach focusing on isolated islets of Langerhans from adult WT mice. We could thereby establish that RSPO1 can also enhance the proliferation of adult murine β cells, which normally display significantly reduced replicating capabilities. Concomitantly, we performed *in vivo* analyses by daily administering RSPO1 to adult WT mice. Quantitative analyses revealed a great increase in β cells in treated animals versus their untreated counterparts. Altogether, these data uncover a significant potential of RSPO1 in stimulating adult β cell neogenesis in a physiological context.

Rspo1 belongs to a family of cysteine-rich secreted proteins mainly known for their role as agonists of the Wnt/β-catenin pathway. In the current study, we demonstrate that RSPO1 induces pancreatic β cell proliferation via the activation of this highly conserved signaling cascade. Of note, previous findings had already showed the importance of the Wnt/β-catenin pathway both in the expansion of the pancreatic tip domain^47^^,^^48^^,^^49^^,^^50^ and in the regeneration of fully differentiated pancreatic endocrine cells.^25^^,^^51^ Also, β-catenin itself has been shown to exhibit both regenerative and insulin secretagogue properties.^51^ Taken together, these discoveries indicate that the proper modulation of the Wnt/β-catenin signaling could represent a reliable alternative for the development of β cell-targeting therapies.

We subsequently wondered whether this capability could be beneficial in a diabetes-like context. To address such question, we first verified whether RSPO1 administration could prevent STZ-induced hyperglycemia. Interestingly, mice pre-treated with RSPO1 showed a significantly lower random-fed glycemia throughout the entire treatment and ultimately exhibited a significantly enhanced glucose tolerance. A similar outcome was observed upon the administration of RSPO1 to readily diabetic animals, with an ameliorated glycemia and an improved glucose tolerance when compared to controls. It should be noted that, for the latter experimental design, the random-fed glycemia curves were not as far apart as expected, mostly due to the controls groups that did not show a sharp increase in glucose levels. We could have used a single administration of a higher dose of STZ, but we chose not to as the glycemia increase could have been much faster than β cell neogenesis and, due to ethical reasons, we would have had to sacrifice massively hyperglycemic animals before any sign of efficacy. In addition, such a quick increase in glycemia/β cell loss would not have been representative of the human disease, which is quite a slow process.^43^ For all these reasons, we chose to rather use NOD mice, which spontaneously develop diabetes as a consequence of insulitis and leukocytic infiltration into pancreatic islets.^52^ The daily administration of RSPO1 prior to the immune attack revealed, once again, a potent protection from hyperglycemia, as well as a strong reduction of the diabetes incidence and a significant increase of the whole β cell mass. This could be explained by either an increased β cell replication or a diminished loss of β cells. While all the aforementioned analyses support the notion of a RSPO1-mediated β cell neogenesis, we could not exclude a certain degree of immune modulation (which would be a great add-on function). Indeed, immunohistochemical analyses of replicating cells in NOD animals are impaired by the overall proliferation of immune cells, rendering the observation of replicating β cells challenging. Interestingly, recent studies have shown that a genetically induced increase of the β cell mass in NOD mice results in beneficial immunomodulation of T cells, reduced β cell apoptosis, and decreased islet infiltration.^53^ It would be of interest to assess whether the β cell proliferation observed in our model is sufficient to induce such modifications.

Noteworthily, Wnt signaling has been often associated with different types of cancer.^54^^,^^55^^,^^56^^,^^57^^,^^58^^,^^59^ Therefore, the use of potent Wnt signaling modulators for clinical application requires considerable safety assessment. However, compounds already approved for diabetes management have been shown to also act as regulators of the Wnt pathway. Among them, exendin-4 has been described not only for its agonistic action on glucagon-like peptide 1 receptor (GLP-1R) but also for its ability to stabilize cytoplasmic β-catenin, thus allowing the activation of several of its target genes.^60^^,^^61^^,^^62^ Importantly, it is worth noting that such a long daily treatment of relatively sensitive NOD mice with RSPO1 did not cause any detectable abnormality, both in live animals and after thorough examination of the extra-pancreatic organs at sacrifice, suggestive of a favorable safety profile for RSPO1. This is in line with a previous phase 1 clinical trial (for alternative applications) first involving 32 (single dose) and later 80 (single and multiple doses) healthy volunteers that did not revealed any abnormality.

Aiming to further improve RSPO1 activities, we designed an optimized RSPO1 protein by coupling it with an FC fragment. Importantly, the results obtained in NOD animals were impressive with a glycemia remaining relatively stable upon weekly administration and a remarkable increase in β cell mass with respect to controls. These results show that FC-RSPO1 can enhance pancreatic β cell proliferation also in a T1D-like context. Obviously, one could wonder whether FC-RSPO1 would reverse the hyperglycemia of readily diabetic NOD mice. However, we would mostly encounter the issue mentioned previously, that is, the development of a very rapid hyperglycemia (it is well known that diabetic NOD mice rapidly develop a strong diabetic state), forcing us to sacrifice the animals for ethical reasons, prior to any sign of efficacy.

Should one consider a possible application of RSPO1 treatment in diabetic patients, one would need to evaluate whether this Wnt agonist could enhance the proliferation of human β cells. Toward this goal, RSPO1 was administered to immunodeficient mice transplanted with human islets. Importantly, a significant augmentation in β cell replication was evidenced following daily treatment. Importantly, such human β cell hyperplasia was associated with a remarkably enhanced human c-peptide secretion, therefore indicating that newly formed human β cells are also metabolically functional.

Taken together, our findings demonstrate that RSPO1 can induce a significant increase in pancreatic β cell proliferation in different *in vitro*, *ex vivo*, and *in vivo* murine models. Importantly, our data suggest a role of this Wnt agonist in efficiently promoting the replication of adult human β cells, which normally exhibit extremely low proliferative capacities. Based on these findings, and on the fact that several Wnt agonists have been already proposed as key molecules for regenerative therapies in other organs/tissues,^63^^,^^64^^,^^65^^,^^66^^,^^67^ we suggest that RSPO1 treatment might represent a very promising approach to restore the β cell pool in diabetic patients.

### Limitations of the study

Despite providing valuable progress in diabetes research, our study still presents some limitations. During our investigation, we resorted to two mutated RSPO1 analogs to demonstrate that preventing binding with either Lgr4 receptor or Znrf3 co-receptor would abolish murine β cell proliferation *in vitro*. However, we are not yet able to provide decisive binding data, because of the difficulties encountered in expressing the extracellular soluble portion of these receptors. We are currently putting all our effort into addressing this key question. Also, another limiting point of our work is the lack of bioavailability data. Toward this goal, we are currently generating customized tools to precisely determine the C_max_, the T_max_, and the bioavailability of our compounds following different routes of administration.

## Resource availability

### Lead contact

Further information and requests for resources should be directed to the lead contact, Patrick Collombat (collombat@unice.fr).

### Materials availability

All proteins (RSPO1 and FC-RSPO1) generated in this study are currently unavailable as they have been entirely used to address reviewers’ questions.

### Data and code availability

This manuscript does not report original code. Any additional information required to reanalyze the data reported in this work is available from the lead contact upon request.

## Acknowledgments

We thank very much C. Wright and M. Sander for the kind gift of antibodies. We are in debt to Metabrain Research for their help in performing some of the experiments mentioned. This work was initially supported by the Juvenile Diabetes Research foundation (#17-2011-16, #2-2010-567, #26-2008-639, and #17-2013-426), the Inserm Avenir program, the 10.13039/501100001677Inserm, the 10.13039/501100000781European Research Council (#StG-2011-281265), the 10.13039/501100002915FRM (#DRC20091217179), the ANR/BMBF (#2009 GENO 105 01/01KU0906), the ‘‘Investments for the Future’’ LABEX SIGNALIFE (#ANR-11-LABX-0028-01), the Max Planck Society, Club Isatis, Mr and Mrs Dorato, Mr and Mrs Peter de Marffy-Mantuano, the Fondation Générale de Santé, and the Foundation Schlumberger pour l’Education et la Recherche. Subsequently, research grants were obtained from DiogenX.

## Author contributions

S.S., T.N., M.P., A.S.-D.-V., H.F., C.A., S.R., B.A., J.B., E.P., V.L., C.T., L.T., J.T., and G.P. contributed to the design and performed the experiments. S.S. and P.C. wrote the manuscript. J.K.-C., F.P., P.B., J.M., A.A., and B.C. contributed to the design of the experiments and the edition of the manuscript.

## Declaration of interests

P.C. is a co-founder and a shareholder of DiogenX. S.S., T.N., C.T., L.E., and L.T. are employees of DiogenX. P.B., J.M., and A.A. are external consultants and shareholders of DiogenX. B.C. is the CEO, co-founder, and shareholder of DiogenX. P.C. and S.S. have a patent related to this work (PCT/EP2021/050289). P.C. and P.B. have a patent related to this work (PCT/EP2022/069925).

## STAR★Methods

### Key resources table

**Table:** 

REAGENT or RESOURCE	SOURCE	IDENTIFIER
**Antibodies**		

FLEX Polyclonal Guinea Pig Anti-Insulin	Dako	Cat# IR00261-2
Rat Monoclonal anti-BrdU antibody	Abcam	Cat# ab6326, RRID:AB_305426
Anti-KI67 antibody, Rabbit monoclonal	Sigma Aldrich	SAB5600050
Goat anti-Guinea Pig IgG Alexa Flour-488	Thermo Fisher	Cat# A-11073, RRID:AB_2534117
Goat anti-Guinea Pig IgG Alexa Flour-594	Thermo Fisher	Cat# A-11076, RRID:AB_2534120
Donkey anti-Rat IgG Alexa Fluor 488	Thermo Fisher	Cat# A-21208, RRID:AB_141709
Donkey anti-Rat IgG Alexa Fluor 594	Thermo Fisher	Cat# A-21209, RRID:AB_2535795
Donkey anti-Rabbit IgG Alexa Fluor 488	Thermo Fisher	Cat# A-21206, RRID:AB_2535792
Donkey anti-Rabbit IgG Alexa Fluor 594	Thermo Fisher	Cat# A-21207, RRID:AB_141637

**Chemicals, peptides, and recombinant proteins**		

DPBS	Fisher Scientific	Cat# 12559069
0,25% sterile-filtered trypsin-EDTA solution	Fisher Scientific	Cat# 25200-056
Heat-inactivated Fetal Calf Serum (FCS)	Sigma Aldrich	Cat# ES-009-B
Penicillin Streptomycin	Gibco	Cat# 15140-122
50μM β-mercaptoethanol	Thermo Fisher	Cat# 31350
DMEM high-glucose	Gibco	Cat# 10569010
MSAB	Merck	Cat# SML1726-5MG
DMSO	Merck	Cat# 20-139
Cardamonin	Merck	Cat# C8249
Bovin Serum Albumin BSA	Sigma Aldrich	Cat# A19418
DMEM low-glucose	Gibco	Cat# 31885-023
Collagenase	Sigma Aldrich	Cat# C7657
1119 Histopaque	Sigma Aldrich	Cat# 1119
1077 Histopaque	Sigma Aldrich	Cat# 1077
HBSS	Gibco	Cat# 14175-053
RPMI-1640	Sigma Aldrich	Cat# R8758
BrdU (5-bromo-2′-désoxyuridine)	Sigma Aldrich	Cat# 19-160
D-(+)-glucose	Sigma Aldrich	Cat# G8270
K3EDTA collection tubes	Starstedt	Cat# 41.3395.005
RNAlater solution	Invitrogen	Cat# AM7024
RNeasy Mini Kit	Qiagen	Cat# 74104
10nM dNTP mix	Invitrogen	Cat# 18427089
50nM Oligo(dT) primer	Invitrogen	Cat# AM5730G
MgCl_2_ (chlorure de magnésium) (25 mM)	Thermo Fisher	Cat# R0971
M-MLV RT Buffer	Invitrogen	Cat# 18057018
RNaseOUT	Invitrogen	Cat# 10777019
Superscript III	Invitrogen	Cat# 18080093
RNase H	Invitrogen	Cat# 18021014
LightCycler® 480 SYBR Green I Master	Roche	Cat# 04887352001
Paraplast	Sigma Aldrich	Cat# P3558
VECTASHIELD HardSet Mounting Medium	Vector	Cat# H-1400-10
Superfrost Plus Adhesion Microscope Slides	Epredia	Cat# J1800AMNZ
Cytospin funnel	Epredia	Cat# A78710013
Cytospin clips	Epredia	Cat# 3120110
Double cytoslides	Epredia	Cat# 5991055
RNAscope™ Probe- Mm-Rspo1	Advanced Cell Diagnostic	Cat# 401991

**Critical commercial assays**		

Mouse CTNNB1/Beta Catenin ELISA	LSBio	Cat# LS-F4891
Steady-Glo® Luciferase Assay System	Promega	Cat# E2510
RNAscope™ Intro Pack 2.5 HD Reagent Kit Brown- Mm	Advanced Cell Diagnostic	Cat# 322371
RNAscope™ 2.5 HD Detection Reagents-BROWN	Advanced Cell Diagnostic	Cat# 322310
Mouse Insulin ELISA	Mercodia	Cat# 10-1247-01
Pierce™ BCA Protein Assay Kits	Thermo Fisher	Cat# 23227

**Experimental models: Cell lines**		

MIN6	AddexBio	Cat# C0018008, RRID:CVCL_0431
266–6	ATCC	Cat# CRL-2151, RRID:CVCL_3481
INS-1 832/13 Rat Insulinoma Cell Line	Merck Millipore	Cat# SCC207
AlphaTC1	ATCC	Cat# CRL-2934, RRID:CVCL_B036
HEK-293 STF	ATCC	Cat# CRL-3249, RRID:CVCL_AQ26

**Experimental mouse models**		

129/SV mice	Chales River	Cat# 287129/SV
NOD mice	Taconic Bioscience	Cat# NOD/MrkTac NOD-F
RAG12N2 mice	Taconic Bioscience	Cat# RAG12-M

**Oligonucleotides**		

Quantitech Primer Assay Gapdh	Qiagen	Cat# QT01658692
Quantitech Primer Assay Rspo1	Qiagen	Cat# QT01554049
Quantitech Primer Assay Lgr4	Qiagen	Cat# QT01065295
Quantitech Primer Assay Lgr6	Qiagen	Cat# QT00296632
Quantitech Primer Assay Lgr5	Qiagen	Cat# QT00123193

**Machines and software**		

Agilent 2100 Bioanalyzer System	Agilent	Cat# 2100
GloMax Explorer luminometer	Promega	Cat# GM3500
Cytospin 4	Epredia	Cat# A78300003
Sunrise BasicTecan spectrophotometer	Tecan	Cat# 30190084
Magellan Software	Tecan	Cat# 30190084
Microtome	Leica	Cat# 2125
Zeiss Axio Imager Z1 Fluo- Microscope	ZEISS	Cat# p1739
Vectra Polaris™ Automated Quantitative Pathology Imaging System	Akoya Bioscience	Cat# CLS143455
HALO/Indica Lab Image Analysis Platform	Indica Lab	https://indicalab.com/halo/
Axiovision software	ZEISS	https://www.micro-shop.zeiss.com/en/us/system/software-axiovision+software-products/1007/
GraphPad Prism 9	GraphPad	https://www.graphpad.com/updates/prism-900-release-notes
LightCycler 480	Roche Life Science	Cat# 05015278001

### Experimental model and study participant details

#### Cell lines

The pancreatic immortalized β-cell lines MIN6 (AddexBio) and Ins1 (ATCC), the α-cell-derived αTC1 (ATCC) and the acinar cell line 266-6 (ATCC) were cultured in 100mm Petri dishes (Eppendorf) in cell line-specific culture media indicated by manufacturer’s instructions and maintained in an humified incubator at 37°C and 5% CO2. Cells were sub-cultured biweekly using the following protocol: plates were first washed with 5 mL of sterile PBS (1X, Gibco) and then incubated for 5 min at 37°C with 3 mL of 0,25% sterile-filtered trypsin-EDTA solution (Gibco). Subsequently, the cells were resuspended in 7mL of complete medium and pelleted via centrifugation at 23°C, 1200 rpm for 5 min. Lastly, supernatant was removed and pellets were resuspended in the cell-specific culture media in order to be plated in new sterile Petri dished. Each cell line was plated in the dilution suggested by the manufacturer and tested periodically for mycoplasma contaminations.

#### Mouse lines

All mouse lines used for this study were housed and manipulated according to the guidelines of the French Regulations for Animal Care, with the approval of the local Ethical Committee. Animals were maintained in a 12-h dark/light cycle and had *ad libitum* access to food and water. Mice were weaned 21 days after birth and fed with chow diet (A03, SAFE). All animals remained in a separated acclimatation room at least 5 days before the experiment. WT 129-SV mice were purchased at Charles River, Non-Obese Diabetic (NOD) and RAG2N12 mice were obtained from Taconic Biosciences.

### Method details

#### Cell number assessment protocol

To assess MIN6 cell number upon incubation with RSPO1 analogs, 80.000 MIN6 cell were initially seeded per well into 12-well plates (5 replicates per condition, *n* = 3) with complete high-glucose (4.5 g/L) DMEM (15% FCS, 1% Penicillin/Streptomycin) and maintained in a humified environment at 37% °C and 5% CO2. Twenty-four hours post seeding, the culture medium was removed and the cells were washed using 1X DPBS. Subsequently, the cells were incubated with different concentrations of RSPO1 analogs (considering a molecular weight of 40kDa for RSPO1, 45kDa for RSPO1 Lgr4^-^ and RSPO1 Znrf3^-^, and about 120kDa for FC-RSPO1 _when accounting for post-translational modifications, diluted into 5% FCS, 1% Pen/Strep DMEM. The cells were incubated exclusively with 5% FCS, 1% Pen/Strep DMEM when used as controls. Following a 24-h incubation, the cells were washed once using 1X DPBS and trypsinized using 0.25% Trypsin-EDTA for 5 min at 37% °C. After inactivation of the trypsin by complete culture medium to each well, MIN6 cells were subsequently transferred into clean 1.5mL Eppendorf tubes and centrifuged at RT for 5 min at 1200rpm. After centrifugation, the supernatant was carefully removed and cell pellets resuspended into clean FCS-free culture medium. Finally, the MIN6 cell counts were assessed manually using a Thoma Chamber.

To assay the impact of the β-catenin inhibitors, MSAB and cardamonin, on cell number, MIN6 cells were seeded, as previously described. Subsequently, MIN6 cells were treated with either medium only, DMSO 0.1%, MSAB 100nM diluted in DMSO, cardamonin 500nM diluted in DMSO and co-treated with either MSAB or cardamonin at the dose previously mentioned and 400nM of RSPO1. Following a 24-h incubation, cells were washed, trypsinized and counted as described previously.

#### MIN6 cell preparation for β-catenin protein level assessment

To determine β-catenin protein levels in presence of RSPO1 by ELISA, 250.000 MIN6 per well were seeded into 6-well plates (5 replicates per condition, *n* = 3) and treated (or not) with 400nM of either RSPO1 or Lgr4^-^ protein as described previously. Following a 3-h incubation, the cells were washed, trypsinized and collected as previously detailed. Following a last centrifugation, the supernatant was carefully removed, cell pellets resuspended in 500μL of sterile 1X PBS, transferred into clean Eppendorf tubes and rapidly frozen in liquid nitrogen before long-term storage at −80C°.

#### HEK Super-TopFlash (STF) reporter assay protocol

HEK293-STF cells (ATCC CRL-3249) were seeded in a 96-well clear bottom cell culture plate at a seeding density of 45.000 cell/well (3 replicates per condition, *n* = 3) in complete DMEM 4.5g/L-glucose (Gibco) supplemented with 10% FBS (Heat inactivated, Gibco) and 1% penicillin/streptomycin and maintained in a humified environment at 37% °C and 5% CO2. Twenty-four hours after seeding, culture medium was removed and the cells were washed using 1X DPBS. Subsequently, HEK293-STF cells were incubated in complete medium at 37% °C and 5% CO2 with different doses (3000- 1000- 333.3- 111- 37- 12.3- 4.1 ng/mL) of either native RSPO1, RSPO1 Lgr4^M^ or RSPO1 Znrf3^M^. A 0.1% BSA solution diluted in PBS was used as negative control for promoter activation. After an additional 24-h incubation, luciferase activity was assessed using a Steady-Go reagent (Promega) following manufacturer’s instructions. Relative Light Units (RLU) for each RSPO1 analog and dose were measured using a GloMax Explorer luminometer.

#### Isolation of murine pancreatic islets for *ex vivo* studies

Murine islets were first isolated from 8-to-10-week-old 129-SV mice by perfusing a solution of 1 mg/ml of collagenase (Sigma Aldrich) diluted in pure Dulbecco’s Modified Eagle’s Medium (DMEM - Gibco) into the main pancreatic duct. Perfused pancreata were then incubated at 37°C for 12 min and subsequently incubated with 10%-FCS DMEM high-glucose to inactivate the collagenase digestive enzymatic activity. Following a series of consecutive centrifugations, islets were purified by tissue fractionation through an Histopaque gradient. Subsequently, murine islets were cultured in RPMI-1640 medium supplemented with 10% FCS and 1% penicillin/streptomycin and maintained overnight into a humified chamber at 37°C and 5% CO2. The following day, 30 islets per well were hand-picked and put into fresh complete culture medium supplemented (or not) with different doses of RSPO1 or FC-RSPO1 (1 replicate per condition, *n* = 3). Fresh medium (with or without RSPO1 analogs) was changed every 24 h. During the last 24 h of treatment, pancreatic islets were co-incubated with 10μM of 5-bromo-2′-dehoxyuridine (BrdU) in order to label cell proliferation. After 72 h islets were transferred into clean Eppendorf tubes, washed 3 times with sterile 1X DPBS by centrifugation (1000rpm, 30 s, 4°C and digested for 3 min in 0.25% Trypsin-EDTA. The enzymatic reaction was stopped by adding completed RPMI medium and by centrifuging at 4°C, 1000rmp for 5 min. Digested islets were finally resuspended into complete RPMI, pipetted into cyto-funnels and cyto-spinned for 5 min at 1650rmp on cytoslides.

#### Human subjects

In accordance with the French authorities and with our Institutional Ethical Committee (« Comité d’Ethique du Center Hospitialier Régional et Universitaire de Lille »), human islets, isolated from brain dead donors were used for research if islets were insufficient in number for clinical transplantation and only if scientific consent was obtained. Islets were transplanted under the kidney capsule of RAG2N12 mice (Taconic Biosciences) as described (Caiazzo et al., 2008), following local ethical committee approval (CEEA2020111).

#### Mouse treatments

For short-term proliferation assessment, 8-week-old 129-SV WT mice (3 mice per condition) were administered intraperitoneally daily for 5 consecutive days with different doses of RSPO1 (0.2, 0.4, and 0.8 mg/kg). In order to label proliferating cells, 5-bromo-2′-dehoxyuridine (BrdU) was injected at a concentration of 100 mg/kg 24 h prior to sacrifice.

NOD mice were treated either daily (RSPO1) or weekly (FC-RSPO1) by intraperitoneal route for 19 weeks. Each protein was diluted freshly in order to obtain a specific concentration (0.4 and 0.8 mg/kg for RSPO1, 2.4 mg/kg for FC-RSPO1) and subsequently administered in a weight-dependent fashion. Glycemia was monitored once a week until week 18 using an Accu-Check reader after blood sampling from the tail vein. After 19 weeks of dosing (or before the animal became diabetic and reached a glycemia >500 mg/dl), animals were sacrificed and their pancreata were then collected for analyses.

Immunodeficient RAG2N12 mice (4 mice per condition) were transplanted with human pancreatic islets (500 IEQ) under the kidney capsule in a sterile environment. The animals underwent a 2-week period to allow recovery, implantation and vascularization of the transplanted cells. After randomization, mice were administered daily by intraperitoneal route with either vehicle, 0.4 or 0.8 mg/kg of RSPO1. Aiming at assessing cell proliferation, mice were co-administered with 5-bromo-2′-dehoxyuridine (BrdU) (1 mg/ml via drinking water) for 7 days prior to sacrifice. BrdU-containing water was dispensed in black feeding bottles, to protect from light, and changed every 2 days until mice sacrifice. After 60 days of treatment, all animals were sacrificed, and the graft-bearing kidneys were isolated for quantitative analyses.

#### Tolerance tests and blood glucose levels measurement

For measurement of basal glycemia and IPGTT, mice were starved for 6 h and subsequently injected intraperitoneally with a weight-dependent dose of D-(+)-glucose (Sigma Aldrich) (2 g/kg). Blood glucose levels were measured right after starvation and/or at the indicated time points after glucose administration using either an Accu-Check or a ONETOUCH Verio glucometer (LifeScan, Inc., CA).

#### Plasma collection

For blood samples collection, mice were anesthetized using isoflurane delivered in oxygen at a flow rate of 1 L/min. Blood was withdrawn from the retro-orbital sinus into K3EDTA blood collection tubes, using glass capillaries. In order to measure basal human c-peptide secretion, blood samples were drawn after 6 h of starvation. To assess glucose-stimulated human c-peptide secretion, an additional blood sampling was performed 15 min after an intraperitoneal injection of weight-dependent dose of D-(+)-glucose (2 g/kg). Whole-blood samples were cooled in iced water right after collection. Plasma was separated by centrifuging at 2000 g at 4C° for 7 min, transferred into pre-cooled tubes and rapidly frozen in liquid nitrogen before long-term storage at −80C°.

#### Enzyme-linked ImmunoSorbent assay (ELISA)

β-catenin protein levels were assessed by ELISA, following manufacturer’s instructions (3 replicates per condition). All reagents and samples were warmed to room temperature before analyses. Absorbance was read using a spectrophotometer (Sunrise BasicTecan, Crailsheim, Germany), complemented by a Tecan’s Magellan data analysis software. Total protein content was also measured using a Thermo Scientific Pierce BCA Protein Assay Kit. Β-catenin concentration was calculated on Microsoft Excel. A second-grade calibration curve was calculated by plotting the known absorbance value of each Calibrator (except Calibrator 0), against the average of the corresponding insulin concentration values. The values obtained were finally normalized on the corresponding total protein content per sample.

In immunodeficient RAG2N12 transplanted mice, human c-peptide blood levels were assessed by ELISA following manufacturer’s instructions (2 replicates per condition).

#### RNA extraction

For total pancreas RNA extraction, a small portion of pancreatic tissue was first dissected and incubated in RNA later solution at 4°C for at least 48h.

For RNA isolation from islets of Langerhans, islets were isolated and cultured as previously described. Following an overnight recovery, islets were hand-picked into a clean Eppendorf tube, centrifuged and resuspended in RLT buffer for long-term storage at −80°C.

RNA from immortalized cell lines (MIN6, INS-1, αTC1 and 266-6) was extracted when cells had reached ∼80–90% confluence. Cells were scraped, collected in a 1.5 mL tube and stored a in RLT buffer for long-term storage at −80°C.

For RNA extraction, samples were disrupted in lysis buffer (RLT buffer from QIAGEN supplemented with 1:1000 β-mercaptoethanol). RNA was then extracted using RNeasy Mini Kit following manufacturer’s instructions. Quantity and integrity (RIN- RNA Integrity Number) were measured using the Agilent 2100 Bioanalyzer System (Agilent Technology). Only samples with RIN ≥7 were kept for cDNA synthesis and gene expression analyses.

#### cDNA synthesis

For retro-transcription, 1μg of RNA was mixed with 1μL of 10mM dNTP, 1μL of 50mM oligoDT and nuclease-free water in a final volume of 10μL. First-strand synthesis was achieved by incubating the obtained mix at 65°C for 5 min. For cDNA synthesis, 10μL synthesis mix (2μL 10x RT buffer; 4μL 25 mM MgCl2; 2μL 0.1 M DTT; 1μL RNase out; 1μL Superscript III - Invitrogen) was added to each sample, successively incubated at 50°C for 50min, at 85° for 5min and finally chilled on ice. A final step of RNaseH incubation at 37°C for 20min was performed in order to remove remaining RNA fragments.

#### Quantitative RT-PCR

RT-qPCR analyses were performed using the QuantiTect SYBR Green RT-PCR kit and QIAGEN primers according to manufacturer’s instructions (3 replicates per condition, *n* = 3). The qPCR program used in the LightCycler 480 instrument (Roche Life Science) was the following: pre-incubation of 10 min at 95°C followed by 45 cycles of amplification (denaturation 10 s 95°C, annealing 45 s 60°C and elongation 1 s 72°C) and 1 cycle of melting curve at 95°C preceded and followed by 10 s of cooling down at 40°C. Each qPCR reaction contained 5 μL of SYBR Green Mastermix, 0.5μL of ready-to-use primer mix, 3μL of nuclease-free water and 1.5μL of cDNA (diluted 1:20 for pancreatic samples and 1:10 for isolated islets and cell lines). The housekeeping gene glyceraldehyde 3-phosphate dehydrogenase (GAPDH) was used as internal control for normalization.

#### Tissue preparation and sectioning

For immunohistochemical analyses, pancreatic tissues were isolated and washed with cold PBS. Subsequently, tissues were fixed into 4% Antigenfix (paraformaldehyde solution pH 7,2–7,4) for 30 min at 4°C. Following fixation, isolated pancreata were dehydrated using increasing concentration of ethanol (50%, 70%, 80%, 90%, and 100%) and then incubated into isopropanol and toluene to remove alcohol residues. Dehydrated organs were then incubated into three consecutive paraffin baths and finally embedded into paraffin using metallic molds. Paraffin blocks were conserved at 4°C prior to sectioning. Thin 6-μm tissue sections were cut using a rotary microtome, put in warm water and subsequently mount on a polarized glass slide (TermoFisher). Slides were first allowed to dry at 42°C for 30 min on a heating plate and then left overnight in pre-warmed oven at 37°C. Slides were conserved at 4°C until deparaffination and immunostaining.

For *in situ* hybridization using the RNAscope (Advanced Cell Diagnostic), pancreatic tissues were promptly isolated, to avoid any RNA degradation due to manipulation, and then incubated into formalin solution (neutral buffered, 10%, Sigma Aldrich) for 16–18 h. All following passages were performed as previously described.

#### Immunohistochemistry

Prior to staining, pancreatic sections were deparaffinized (3 × 3min in xylene), rehydrated into decreasing concentration of alcohol (2 × 5min 100% ethanol, 1 × 5 min 80% ethanol, 1 × 5 min 60% ethanol, 1 × 5 min 30% ethanol) and washed twice with ddH2O. In order to reveal epitopes that might have been masked during tissue preparation, slides were boiled in a pressure cooker for 1 min in 1.6 L of ddH2O and 15 mL of Unmasking Solution (citric acid based, pH 6, Vector Laboratories). Slides were successively cooled down with fresh tap water for at least 30 min. Tissues were washed 3 × 5 min in PBS and then incubated at room temperature (RT) for 45 min with a blocking solution (PBS, 10% fetal calf serum- FCS) in order to avoid non-specific antibody binding. After blocking, slides were incubated overnight at 4°C with primary antibodies, treated with a blocking solution (PBS 10% FCS) and finally incubated overnight at 4°C with primary antibodies diluted into blocking solution. To remove primary antibodies excesses, slides were washed 3 × 5 min with clean PBS and then incubated for 45 min at RT with secondary antibodies, diluted 1:1000 into blocking solution. Finally, pancreatic sections were washed 3 × 5min with clean PBS, mounted with coverslips using a hard-set Vectashield HardSet Mounting Medium containing 4′, 6-diamidino- 2-phenylindole (DAPI) for nuclear staining, dried and kept at 4°C until image acquisition.

For *ex vivo* islet studies, cytoslides were fixed for 30 min using 3,7% PFA, washed 3 times with 1X PBS and permeabilized using a Triton 100 × 0.5% in BSA 0.2%. Antigen retrieval was then performed by boiling the slides for 20 min into 1.6 L of ddH2O and 15 mL of Unmasking Solution (citric acid based, pH 6, Vector Laboratories). To prevent non-specific binding, slides were incubated for 45 min with a PBS-5% BSA blocking solution before immunostaining. Subsequently, sections were incubated O.N at 4°C with primary antibodies in blocking buffer. The day after, slides were washed 3 times for 5 min with clean 1X PBS and then incubated for 45 min at RT with secondary antibodies diluted 1:1000 in blocking buffer. Finally, stained slides were washed 3 times for 5 min with clean 1X PBS, mounted with coverslips using a hard-set Vectashield HardSet Mounting Medium containing 4′, 6-diamidino- 2-phenylindole (DAPI) for nuclear staining, dried and kept at 4°C until image acquisition.

#### RNAscope

*In situ* RNA detection of *Rspo1* transcripts was performed using RNAscope. Sample pretreatment, probe hybridization and signal amplification and detection were carried according to manufacturer’s instructions. Finally, slides were mounted with coverslips using the hard-set Vectashield Mounting Medium and stored at RT until image acquisition.

### Quantification and statistical analysis

#### Quantification analyses

Single images were acquired using ZEISS Axiomanager Z1, equipped with the appropriate filter sets and a monochrome camera, and processed with Axiovision software from ZEISS. Whole-section mosaics were obtained using Vectra Polaris Automated Quantitative Pathology Imaging System. The entire pancreata of at least 5 mice per condition were serially cut, applied on glass slides and every 5 to 10 pancreatic sections were subsequently processed for immunohistochemistry. For hormone quantification, widefield multi-channels tile scan images treatment and analyses were performed using Fiji semi-automated macro. Specifically, the whole pancreas slice surface was determined and measured using a low intensity-based threshold on the insulin labeling channel. Islets were subsequently segmented and quantified on insulin labeling using a high intensity threshold. The density of islets per pancreatic section was obtained by reporting the number of islets to the section area. Hormone quantification on mosaic images was obtained using the HALO/Indica Lab Image Analysis Platform for image quantification. Endocrine cell proliferation was quantified using Axiovision software from ZEISS. Proliferating cells (BrdU+ or Ki67+) were manually counted on each section and normalized on the corresponding total surface (measured in μm2).

#### Data analyses and statistics

All values are represented as mean ± SEM of sets of data. Data were analyzed using GraphPad Prism 9 software. Normal distribution was examined using the D’Agostino-Pearson omnibus normality test. Subsequently, appropriate statistical tests were adopted. For pairwise comparisons, parametric (if normality test was passed) or non-parametric (in case of normality test not passed) T-test was performed. For analyses on a single independent variable parametric (if normality test was passed) or non-parametric (in case of normality test not passed) one-way ANOVA was adopted. For multiple comparisons on independent variables a two-way ANOVA was applied using a Bonferroni’s adjustment for significance threshold correction. Results were considered significant if *p* < 0.0001 (∗∗∗∗), *p* < 0.001 (∗∗∗), *p* < 0.01 (∗∗) and *p* < 0.05 (∗).

## Supplemental information

Document S1. Figures S1–S6

Document S2. Article plus supplemental information
