# Supplementary material for: RSPO1, a potent inducer of pancreatic β cell neogenesis
Source: Cell Rep Med. 2025 May 7;6(5):102126. doi: 10.1016/j.xcrm.2025.102126 (PMC12147903; doi:10.1016/j.xcrm.2025.102126)
Supplement: Document S1. Figures S1–S6 [file mmc1.pdf]

Cell Reports Medicine, Volume *6*

Supplemental information

RSPO1, a potent inducer

# of pancreatic β cell neogenesis

Serena Silvano, Tiziana Napolitano, Magali Plaisant, Anette Sousa-De-Veiga, Hugo Fofo, Chaïma Ayachi, Benoit Allegrini, Samah Rekima, Estelle Pichery, Jérôme Becam, Valentin Lepage, Caroline Treins, Laura Etasse, Loan Tran, Julien Thévenet, Gianni Pasquetti, Julie Kerr-Conte, François Pattou, Paolo Botti, Arduino Arduini, Jacques Mizrahi, Benjamin Charles, and Patrick Collombat

SUPPLEMENTAL FIGURES AND FIGURE LEGENDS

Silvano *et al.*, 2025 - Figure S1

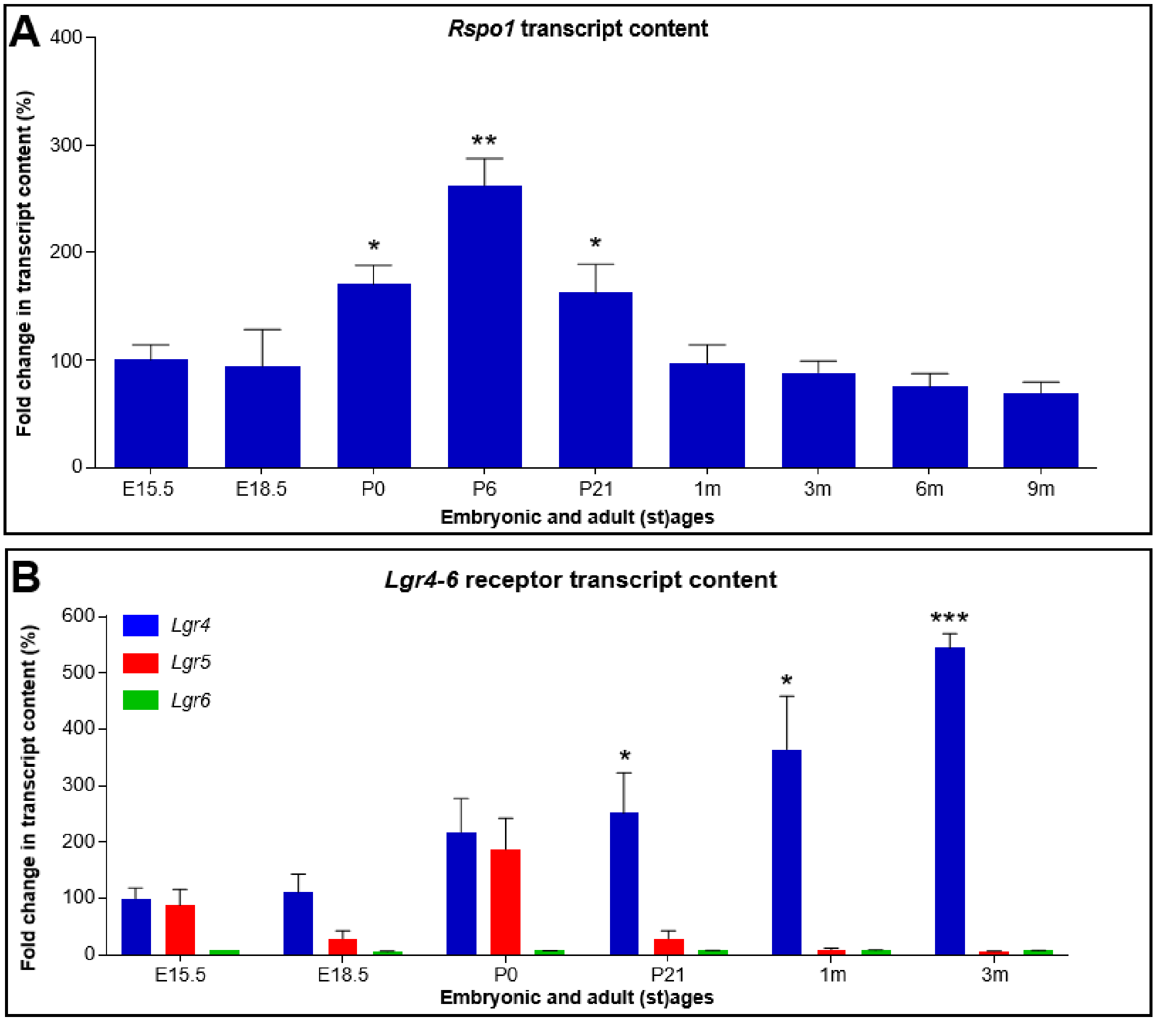

Figure S1. Monitoring of pancreatic *Rspo1*, *Lgr4*, *Lgr5,* and *Lgr6* expression levels from late embryogenesis and thereafter. Related to Figure 1. Quantification of the expression levels of *Rspo1* (**A**), *Lgr4, Lgr5, and Lgr6* (**B**) assessed by RT-qPCR using mRNAs extracted from WT pancreata at different embryonic stages and adult ages. All data shown in **A-B** represent mean ± SEM of n=5. Results were considered significant if $p < 0.0001$ (****), $p < 0.001$ (***), $p < 0.01$ (**) and $p < 0.05$ (*) following a one-way ANOVA.

Silvano *et al.*, 2025 - Figure S2

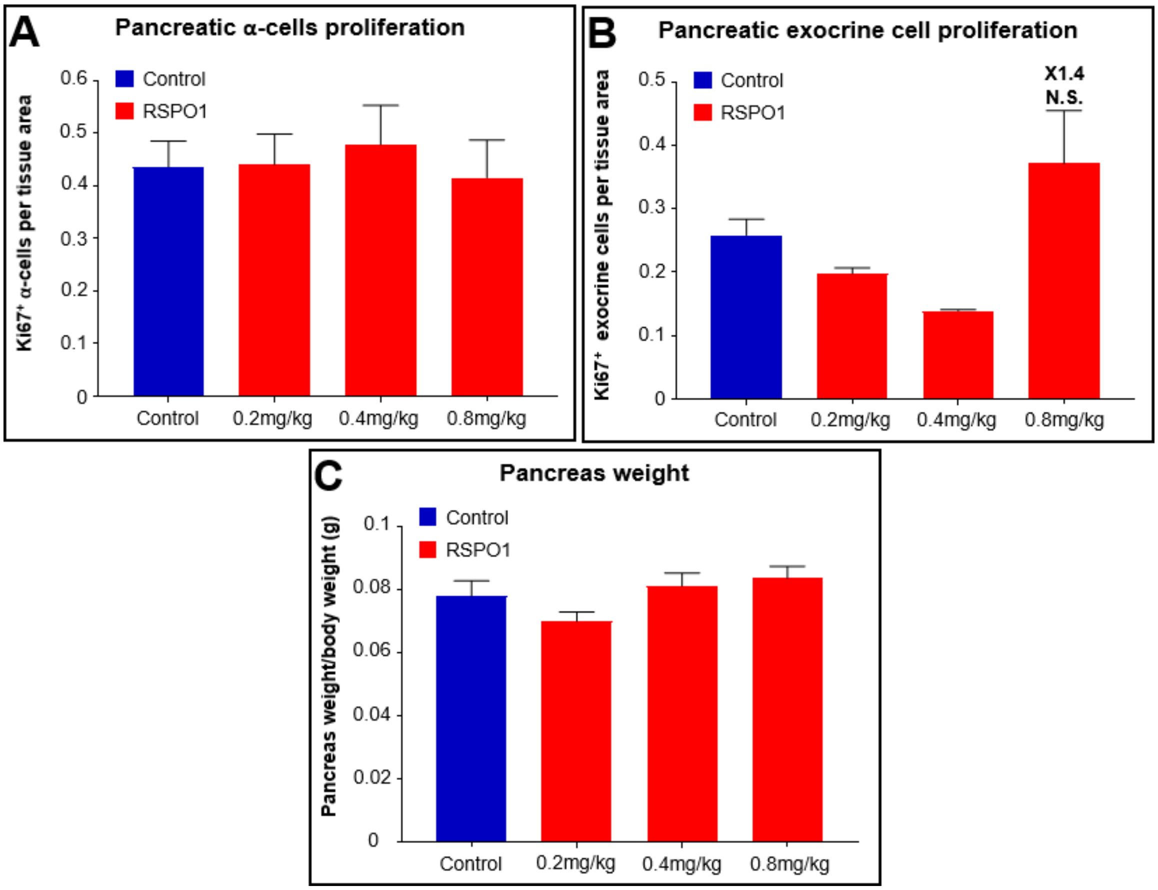

Figure S2. RSPO1 does not induce α-cell nor exocrine cell proliferation. Related to Figure 2. (A-B) Quantitative assessment of $Ki67^+$ α-cells per islet **(A)** and exocrine cells per tissue area **(B)** following 5 consecutive intraperitoneal RSPO1 administrations. **(C)** Average pancreas weight normalized on the body weight of mice administered for 5 consecutive days with either different doses of RSPO1 or PBS (controls). Data shown represent mean ± SEM of n=5. Results were considered significant if $p < 0.0001$ (****), $p < 0.001$ (***), $p < 0.01$ (**) and $p < 0.05$ (*) following a one-way ANOVA.

Silvano *et al.*, 2025 - Figure S3

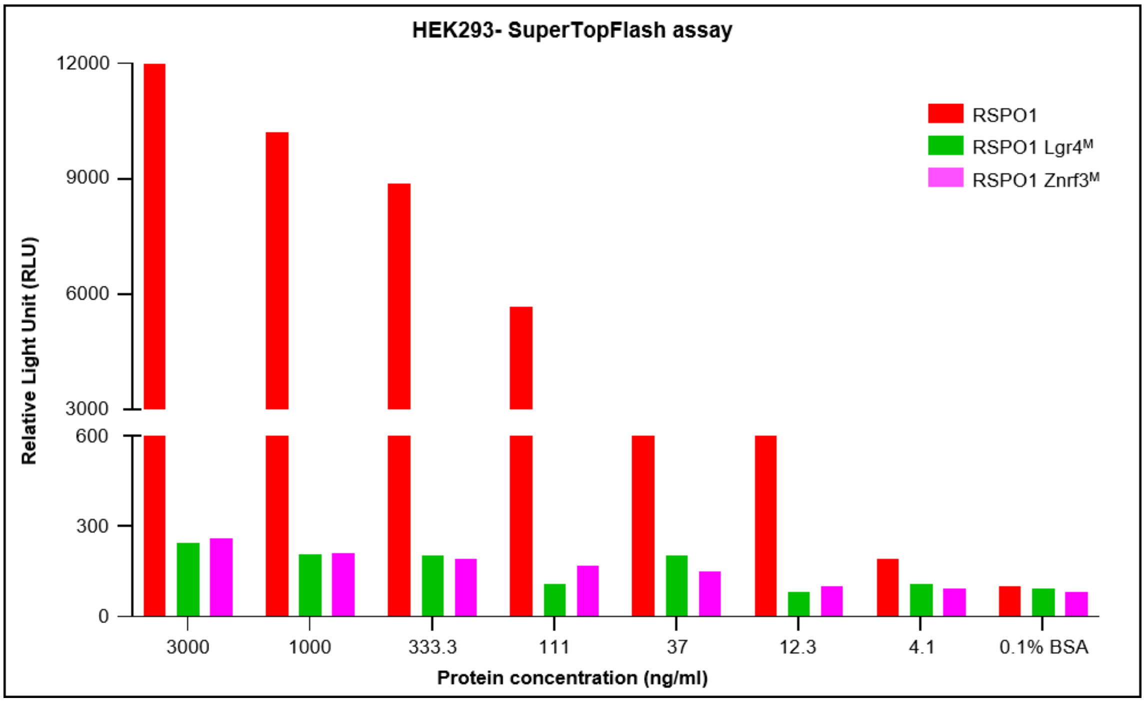

Figure S3. RSPO1 Lgr4^{M} and RSPO1 Znrf3^{M} do not activate the HEK293-STF reporter in a TopFlash assay. Related to Figure 3. Effect of native RSPO1, RSPO1 Lgr4^{M} and RSPO1 Znrf3^{M} on HEK293-STF showing no activity of the two mutant proteins on the LEF/TCF promoter at any of the doses tested as compared to native RSPO1. Experiments were repeated in duplicates and reporter activity was expressed in Relative Light Units (RLU).

Silvano *et al.*, 2025 - Figure S4

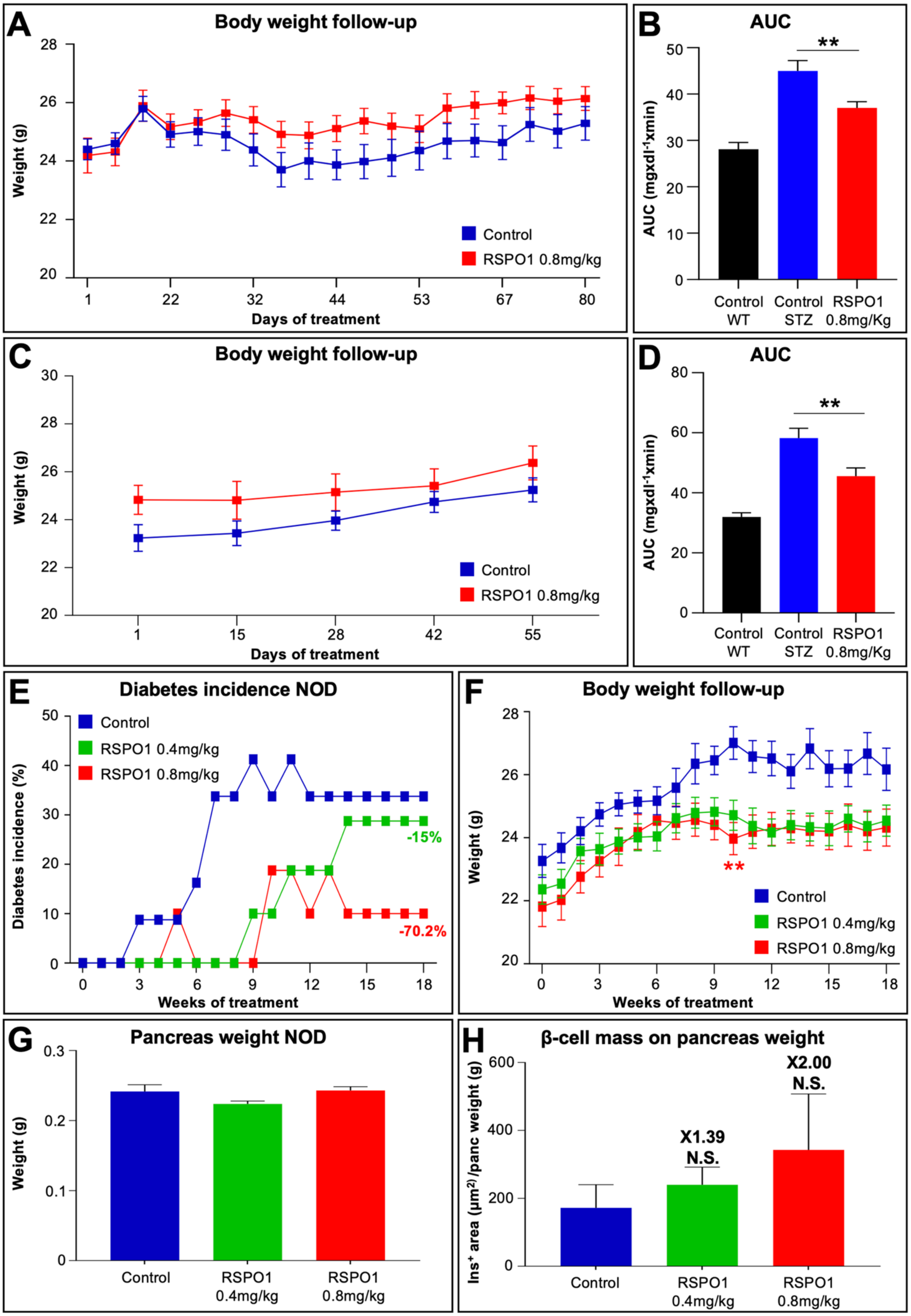

Figure S4. Effect of RSPO1 administration on diverse physiological parameters in two different T1D models. Related to Figure 4. (A) Body weight follow-up of WT mice administered intraperitoneally for 80 consecutive days with either saline or 0.8mg/kg of RSPO1 and co-treated with 50mg/kg of streptozotocin from day 16 to day 18 (n=10). **(B)** Area under the curve (AUC) of IPGTTs performed on WT mice injected intraperitoneally for 80 consecutive days with either saline or 0.8mg/kg of RSPO1 and co-treated with 50mg/kg of STZ from day 16 to day 18 (n=10).

(C) Body weight measurements of WT mice first administered for 3 consecutive days (1-3) with 50mg/kg of STZ and subsequently injected daily with either saline or RSPO1 at 0.8mg/kg, once their glycemia reached 250mg/dl (approximately at day 7) (n=10). **(D)** Area under the curve (AUC) of IPGTTs performed on WT mice first injected for 3 consecutive days (1-3) with 50mg/kg of STZ and then administered daily with either vehicle or RSPO1 at 0.8mg/kg (n=10). **(E)** Diabetes incidence of NOD mice treated with either 0.4, or 0.8mg/kg of RSPO1 or saline. Diabetes incidence was calculated using the following formula: (number of diabetic mice (glycemia> 250mg/dl)*100)/total number of mice per experimental group) **(F)** Weekly monitoring of the body weight of NOD females injected daily intraperitoneally for 18 weeks with either saline, 0.4, or 0.8mg/kg of RSPO1 (n=15 in control group, n= 10 to 13 in treated groups).

(**G**) Average of the pancreas weight of NOD mice treated daily with either saline, 0.4, or 0.8mg/kg of RSPO1 for 18 consecutive weeks. **(H)** Quantification of the whole β-cell mass in NOD mice administered daily with either 0.4mg/kg, 0.8mg/kg of native RSPO1 or saline. For this calculation the insulin$^+$ area of each section was normalized on the pancreas weight of the corresponding animal (n=15 in control group, n= 10 to 13 in treated groups). All data shown represent mean ± SEM. Results were considered significant if $p < 0.0001$ (****), $p < 0.001$ (***), $p < 0.01$ (**) and $p <0.05$ (*) following a 2way ANOVA (**A-C-F**), a one-way ANOVA (**B-D**) or a Kruskal-Wallis comparison test (**H**).

Silvano *et al.*, 2025 - Figure S5

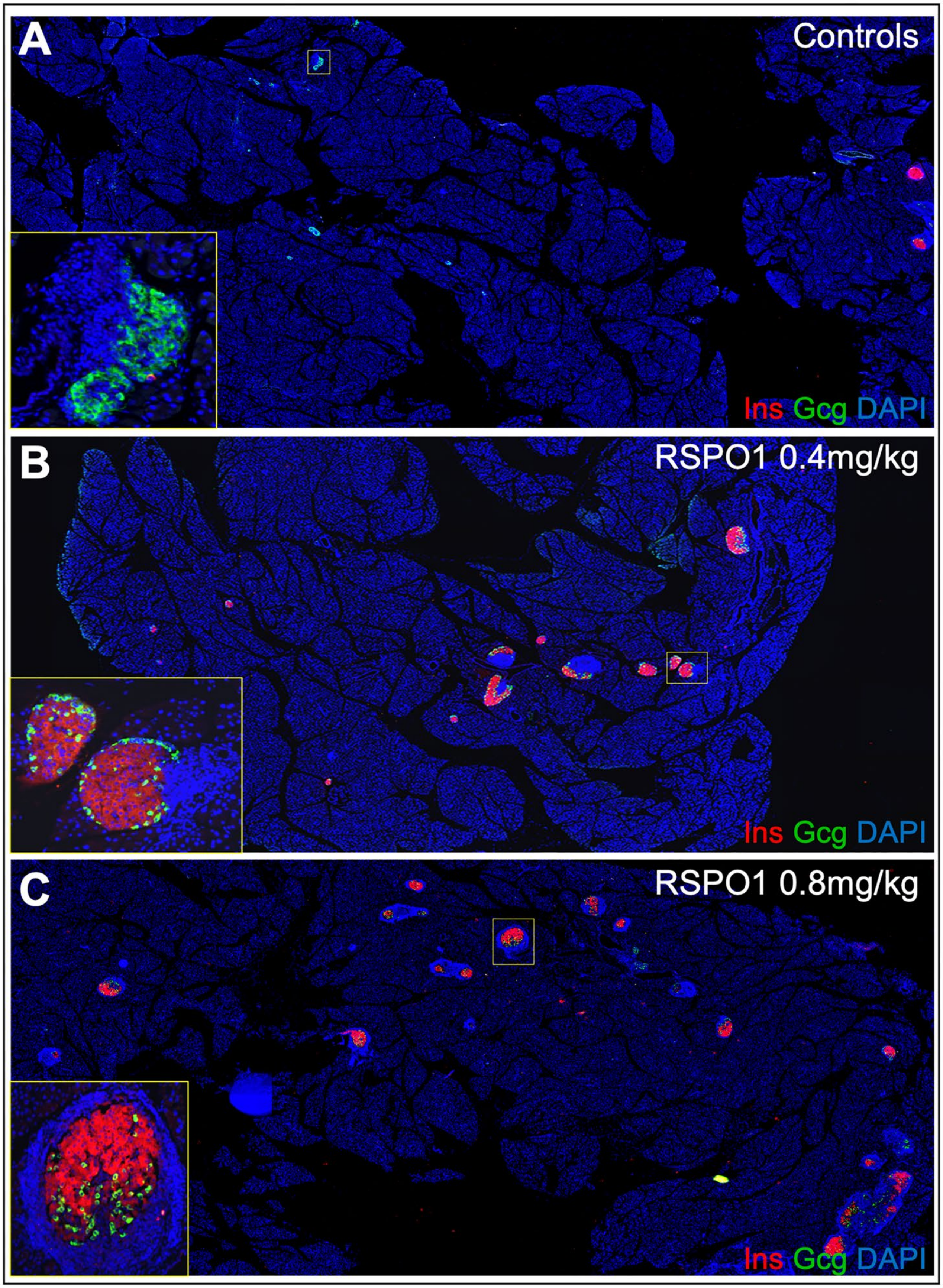

Figure S5. Assessment of pancreatic β-cell mass in NOD mice treated with RSPO1 at 0.4 and 0.8mg/kg. Related to Figure 4. Immunohistochemical analyses to assess the whole β-cell mass of NOD mice treated daily for 18 consecutive weeks with either PBS **(A)**, 0.4 **(B)** or 0.8mg/Kg of RSPO1 **(C)**. Pancreatic sections of the three different groups were stained using an anti-insulin antibody (red), an antibody recognizing glucagon (green) and with DAPI to label all nuclei.

Silvano *et al.*, 2025 - Figure S6

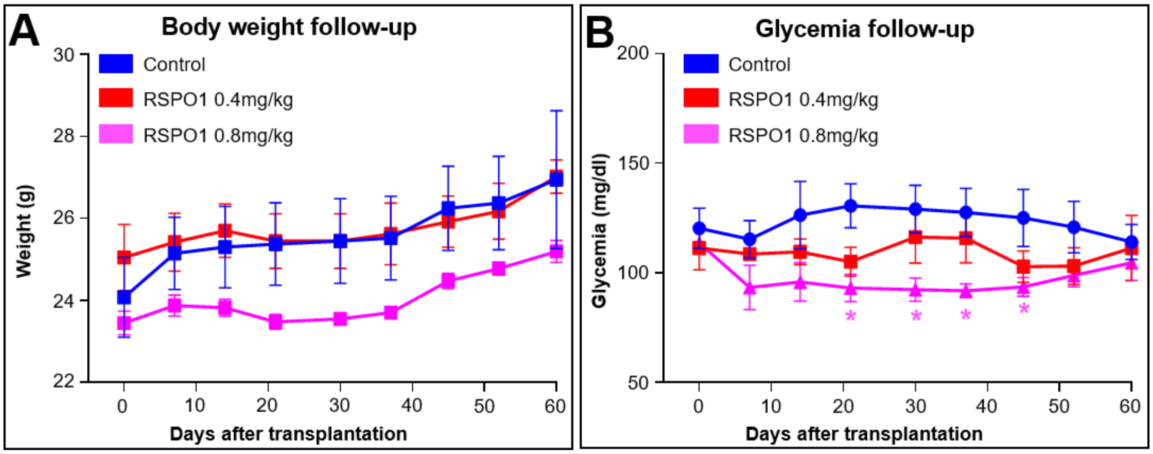

Figure S6. Monitoring of physiological parameters in transplanted RAG12N2 immunocompromised mice. Related to Figure 6. Body weight (**A**) and glycemia (**B**) follow-up of RAG12N2 mice before (day 0) and after the transplantation of human islets under the kidney capsule. All data shown represent mean ± SEM of n=4. Results were considered significant if $p < 0.0001$ (****), $p < 0.001$ (***), $p < 0.01$ (**) and $p < 0.05$ (*) following a 2way ANOVA.
